# Computer‐extracted features of nuclear morphology in hematoxylin and eosin images distinguish stage II and IV colon tumors

**DOI:** 10.1002/path.5864

**Published:** 2022-02-22

**Authors:** Neeraj Kumar, Ruchika Verma, Chuheng Chen, Cheng Lu, Pingfu Fu, Joseph Willis, Anant Madabhushi

**Affiliations:** ^1^ Department of Computing Science University of Alberta and Alberta Machine Intelligence Institute Edmonton Canada; ^2^ Department of Biomedical Engineering Case Western Reserve University Cleveland OH USA; ^3^ Department of Population and Quantitative Health Sciences Case Western Reserve University Cleveland OH USA; ^4^ Department of Pathology Case Western Reserve University Cleveland OH USA; ^5^ University Hospitals Cleveland Medical Center Cleveland OH USA; ^6^ Louis Stokes Cleveland Veterans Administration Medical Center Cleveland OH USA

**Keywords:** computational pathology, quantitative histomorphometric image analysis, colon cancer, hematogenous spread, peritoneal spread

## Abstract

We assessed the utility of quantitative features of colon cancer nuclei, extracted from digitized hematoxylin and eosin‐stained whole slide images (WSIs), to distinguish between stage II and stage IV colon cancers. Our discovery cohort comprised 100 stage II and stage IV colon cancer cases sourced from the University Hospitals Cleveland Medical Center (UHCMC). We performed initial (independent) model validation on 51 (143) stage II and 79 (54) stage IV colon cancer cases from UHCMC (The Cancer Genome Atlas's Colon Adenocarcinoma, TCGA‐COAD, cohort). Our approach comprised the following steps: (1) a fully convolutional deep neural network with VGG‐18 architecture was trained to locate cancer on WSIs; (2) another deep‐learning model based on Mask‐RCNN with Resnet‐50 architecture was used to segment all nuclei from within the identified cancer region; (3) a total of 26 641 quantitative morphometric features pertaining to nuclear shape, size, and texture were extracted from within and outside tumor nuclei; (4) a random forest classifier was trained to distinguish between stage II and stage IV colon cancers using the five most discriminatory features selected by the Wilcoxon rank‐sum test. Our trained classifier using these top five features yielded an AUC of 0.81 and 0.78, respectively, on the held‐out cases in the UHCMC and TCGA validation sets. For 197 TCGA‐COAD cases, the Cox proportional hazards model yielded a hazard ratio of 2.20 (95% CI 1.24–3.88) with a concordance index of 0.71, using only the top five features for risk stratification of overall survival. The Kaplan–Meier estimate also showed statistically significant separation between the low‐risk and high‐risk patients, with a log‐rank *P* value of 0.0097. Finally, unsupervised clustering of the top five features revealed that stage IV colon cancers with peritoneal spread were morphologically more similar to stage II colon cancers with no long‐term metastases than to stage IV colon cancers with hematogenous spread. © 2022 The Authors. *The Journal of Pathology* published by John Wiley & Sons Ltd on behalf of The Pathological Society of Great Britain and Ireland.

## Introduction

A critical unmet need in gastrointestinal oncology is to identify colorectal cancer patients at high risk of tumor recurrence after potentially curative surgery [1, 2]. Although AJCC tumor, node, metastasis (TNM) staging remains the bedrock of patient risk stratification [3], it is widely recognized that better systems are needed. This is highlighted by the fact that up to 25% of stage II colon cancer (CC) patients will develop distant metastases within a 10‐year period [4, 5, 6].

Multiple morphological and molecular parameters are predictive of patient outcomes, including poor differentiation, lymphovascular or perineural invasion, and tumor infiltration pattern [7, 8, 9]. Tumor‐infiltrating lymphocyte density and tumor budding, along with other parameters, have also been identified as promising prognostic features in CC [10, 11, 12, 13, 14, 15, 16, 17]. However, problems pertaining to the lack of workable quantitative classification schemes and inter‐pathologist reproducibility make implementation of morphology‐based patient risk stratification difficult to achieve [13, 18, 19, 20, 21, 22]. Numerous studies have identified molecular markers that are associated with CC patient outcomes [23, 24]. However, apart from mismatch repair/microsatellite status and *BRAF* mutation status in microsatellite‐stable CC, none of these markers have proven robust enough to warrant inclusion into standard clinical care pathways [24].

In the past decade, the availability of digital whole slide imaging (WSI) has paved the way for computerized assessment of tissue pathology through quantitative histomorphometric analysis (QHA) for disease characterization. QHA uses computer‐extracted features to decrypt sub‐visual differences of tumor morphology in digital tissue images. Recently, several deep‐learning‐based approaches composed of multiple processing layers to learn feature representations with multiple levels of abstractions [25] have been developed to learn the feature representations for QHA in both supervised [26] and unsupervised [27] approaches from WSI. Such approaches have been widely used for predicting patient outcomes, mutational profiles, and microsatellite instability across tumors of various organs [28, 29, 30, 31, 32, 33]. An alternate, but more interpretable, approach is to use explainable handcrafted features that relate to specific structures in pathology images, e.g. cancer nuclei for predicting disease outcomes [34, 35]. QHA with handcrafted features has demonstrated an ability to reproducibly define patient outcomes in multiple cancer systems [21, 36, 37], to correlate with molecular classifications [36, 38], and tumor–host responses [36]. Recently, it has been shown that nuclear architecture including nuclear shape, size, and texture is useful in cancer diagnosis, grading, prognostication, and prediction of response to therapy in a number of cancer types [35, 39, 40, 41, 42, 43].

Approximately 25% of CC patients will have distant disease at initial diagnosis and about 50% of all CC patients will develop distant metastases – most commonly to the liver. These are assumed to be via venous hematogenous spread [44]. The peritoneal cavity is the third most common site of metastases – after the lung – and is likely to be caused by direct peritoneal extension of the primary cancer in the majority of cases. Apart from definition of involvement of the visceral peritoneum, there are no known morphological or molecular features that separate primary colon cancers with hematogenous metastases from those with peritoneal metastases.

In this study, we investigated the hypothesis that stage II CCs of standard histologic type with no evidence of long‐term recurrence are morphologically distinguishable through QHA from stage IV CCs which present with hematogenously derived metastases (typically to the liver and lung). We also investigated whether CCs that recurred via intraperitoneal spread had different QHA profiles to those with hematogenous dissemination. QHA results were generated via ‘handcrafted’ computational image analyses to evaluate the role of CC nuclear shape and texture features from a pathological spectrum of *N* = 527 WSIs of stage II and stage IV CCs, with 200 cases in a discovery set and 327 cases in a validation set.

## Materials and methods

### Brief overview

**Figure 1:**
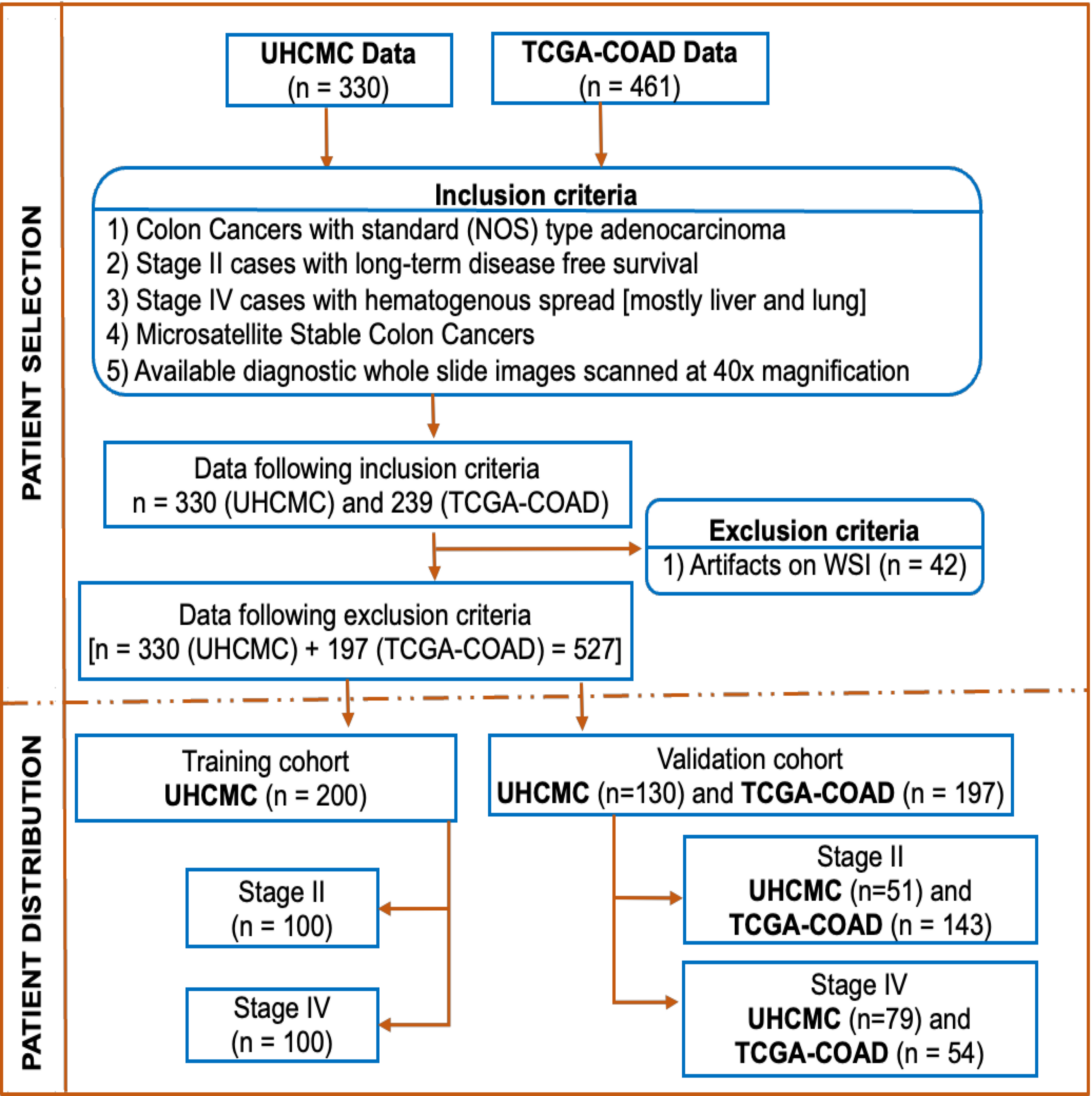
Patient inclusion criteria and distribution of cases in the training and validation cohorts.

The main steps adopted in our approach were as follows. First, WSIs of hematoxylin and eosin (H&E)‐stained surgical pathology slides of formalin‐fixed, paraffin‐embedded (FFPE) CC specimens were obtained. Second, a deep convolutional neural network was trained to separate tumor regions from non‐tumor regions. Third, segmentation of nuclei was performed using another deep‐learning algorithm trained on a publicly available dataset containing 29 000 manually annotated nuclei, spanning several organs, patients, disease states, and tissue source sites [45]. Fourth, we extracted several features pertaining to architecture, shape, texture, and spatial arrangement of tumor nuclei. These were used in conjunction with our machine learning classifier to distinguish between stage II and stage IV CC and independently validated on the TCGA‐COAD cohort cases. Finally, we interrogated the nuclear features of stage IV CC with peritoneal metastases and compared these with both stage II CC and stage IV CC with hematogenous spread through unsupervised clustering (Figure 1).

### Dataset description

Figure 1 shows a flow chart of the patient inclusion and exclusion criteria for this study. We obtained H&E WSIs for 151 stage II CCs with long‐term disease‐free survival and 179 stage IV CCs with hematogenous spread (mostly the liver and lung) from University Hospitals Cleveland Medical Center (UHCMC). The UHCMC cases were divided into two subsets: a training dataset, S_tr,_ of 100 stage II and 100 stage IV CCs, and a UHCMC validation set, S_v_, containing 51 stage II and 79 stage IV CCs for performance evaluation of the trained model. Another independent validation set, S_t_, from the TCGA‐COAD cohort with WSIs of 143 stage II and 54 stage IV CCs (FFPEs) was used for external performance evaluation. All stage IV CCs included in the study had evidence of hematogenous metastases. An expert gastrointestinal pathologist (JW) reviewed all the UHCMC and TCGA‐COAD cases, and only CCs with standard (not otherwise specified)‐type adenocarcinoma were included in the study. All the UHCMC cases included in this study were scanned at 40× microscopic magnification on a Ventana iScan HT scanner (Roche, Nutley, NJ, USA). An additional 28 UHCMC stage IV CCs (FFPEs) with peritoneal metastases (21 without documented hematogenous metastases) were added to the validation set (S_v_) to assess the efficacy of the trained classifier to distinguish between stage IV CC with peritoneal metastases and stage IV CC with hematogenous metastases. The number of WSIs used in the training, validation, and test sets is also presented in supplementary material, Table S1.

### Tumor segmentation

**Figure 2:**
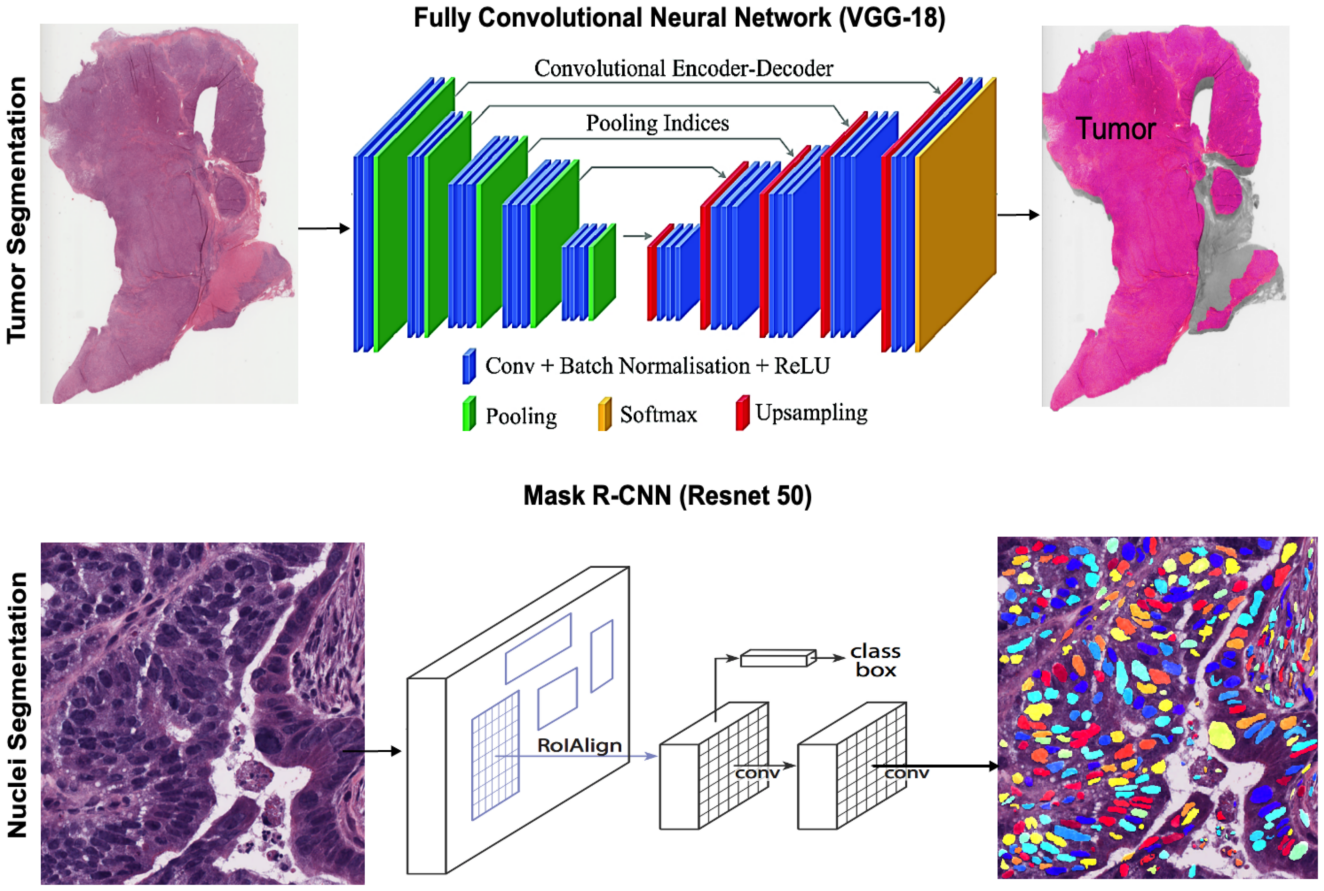
Illustration of the tumor and nucleus segmentation modules.

To localize the tumor region within the WSI, a fully convolutional neural network (FCN) with VGG‐18 architecture [46] was employed for tumor segmentation (see supplementary material, Table S2 for hyperparameter settings). Vahadane *et al*'s [47] approach for color normalization was applied to each WSI before feeding it to the tumor segmentation network. For training, 50 images per class (stage II and stage IV CC) were randomly selected from S_tr_ and an expert pathologist performed manual tumor annotations. For validation, an expert pathologist marked the tumor regions in 50 randomly selected cases from the UHCMC validation set S_v_. Comparison of the ground‐truth (pathologist marked) and algorithm‐computed tumor segmentation masks for these 50 cases yielded an average intersection‐over‐union (IoU) value of 0.81. The tumor segmentation network predicted the probability of each input patch (size 512 × 512 pixels) belonging to the tumor or not, and cross‐entropy loss function for binary classification (tumor versus non‐tumor) was employed during the training phase. An illustration of the tumor segmentation convolutional neural network is shown in the top row of Figure 2. The trained model was then applied to segment tumor regions in both UHCMC (S_v_) and TCGA‐COAD (S_t_) cases used in this study.

### Segmentation of nuclei

Following tumor segmentation, another convolutional neural network to segment nuclei within the tumor region was trained. The training and validation data for segmentation of nuclei were obtained from an international competition on multi‐organ nuclei segmentation – MoNuSeg [45]. The advantage of using the MoNuSeg dataset was that it was sourced from several organs and covered a wide range of nucleus morphologies across cancer types and stages. The nucleus segmentation module used in the current project obtained an average aggregated Jaccard index (AJI) of 0.70 on the challenge's validation dataset – which is on par with the winning entry of the challenge [45]. Although details of the nucleus segmentation module appear in the Supplementary materials and methods, an illustration of our Mask R‐CNN [48]‐based nucleus segmentation is shown in the bottom row of Figure 2.

### Feature extraction

A total of 26 641 nuclear features were extracted from the segmented tumor nuclei per 1000 × 1000 patch, where each patch contained around 30 (±10) nuclei on average. The quantified nuclear features extracted were size, shape, texture, orientation, architecture, and spatial organization. Shape features included invariant moment, Fourier descriptor, and length/width ratios. Nuclear texture was captured using the Haralick texture features. We also computed cell cluster graphs (CCGs) to extract the local neighborhood‐based basic shape features as previously described [43]. Disorder in the orientation of tumor nuclei was captured using cell graph tensors (CGTs) defined over the local CCG [49]. A more detailed description of the extracted nuclear features is provided in Supplementary materials and methods.

### Feature selection and classifier construction

The most relevant nuclear features for discriminating between stage II and stage IV CC were identified using the Wilcoxon rank‐sum test (WRST). We limited the number of candidate features to 5 to avoid dimensionality and overfitting in the subsequent classifier. The top five WRST‐identified features were then used to construct a random forest (RF) classifier to distinguish between stage II and stage IV CC. The RF classifier was trained on the UHCMC training data (see Dataset description) by keeping 80% of the data for training and the remaining 20% for initial model evaluation. The classifier was trained on a per‐patch basis, where patient level classification decision (stage II versus stage IV) was obtained by tallying the number of patches identified as stage II or stage IV CC and classifying the patient based on which stage had the majority. This voting method was used to classify the features corresponding to each patient's known tumor stage in the training set. The per‐patient patch voting accuracy was defined as the percentage of patients whose tumor stage was classified correctly using this method.

### Statistical analyses

We evaluated the classification accuracy of the dichotomous machine learning classifier, for stage II versus stage IV classification, in terms of precision‐recall receiver operating characteristics area under the curve (ROC‐AUC). We also assessed the distribution of the top five most relevant and discriminative tumor nuclear features using violin plots. A supplementary survival analysis was performed on overall survival data available for TCGA‐COAD using Kaplan–Meier plots and a Cox proportional hazards model. No survival analysis was carried out for UHCMC cases, as survival information was not available for those patients.

We also employed uniform manifold approximation and projection (UMAP) embeddings and violin plots to illustrate the clustering of stage II, peritoneal stage IV, and hematogenous stage IV CC in the nuclear feature space to highlight the separation between these cases according to the top discriminatory features obtained from the supervised classification analysis. We also assessed the groupings of stage II and stage IV CC with both peritoneal and hematogenous metastases using an unsupervised hierarchical clustering‐based heatmap.

To assess the effectiveness of the trained models, the model with the highest performance in terms of AUC was tested on an external validation set (TCGA‐COAD cohort). Models were trained over the entire primary cohort (UHCMC training set) before being applied, without any retraining to the external validation set.

## Results

### Patient characteristics

The data in the UHCMC cohort comprised 53% women and 47% men, with an average age of 69 years. Around 72% of patients were Caucasian, while the rest were African Americans. The TCGA‐COAD cohort consisted of 48% women and 52% men, with an average age of 66 years, who were predominantly Caucasian. The tumors of all but one patient included in this study were microsatellite‐stable.

### Experiment 1: evaluating the ability of nuclear histomorphometric features to distinguish stage II from stage IV colon cancers

Following feature extraction, the Wilcoxon rank‐sum test (WRST) was employed to select the top five discriminatory features in the UHCMC training set (S_tr_). The selected feature set comprised (1) nuclear area, (2) nuclear perimeter, (3) major‐axis length of nuclei, (4) variance of nuclear contrast, and (5) entropy of nuclear orientation as the most statistically significant discriminatory features. An RF classier with these features on the UHCMC validation set (S_v_) and the TCGA‐COAD independent validation set (S_t_) yielded AUCs of 0.81 and 0.78. The AUC‐ROC plots of the combined model with the top five discriminatory features are shown in supplementary material, Figure S1.

**Figure 3:**
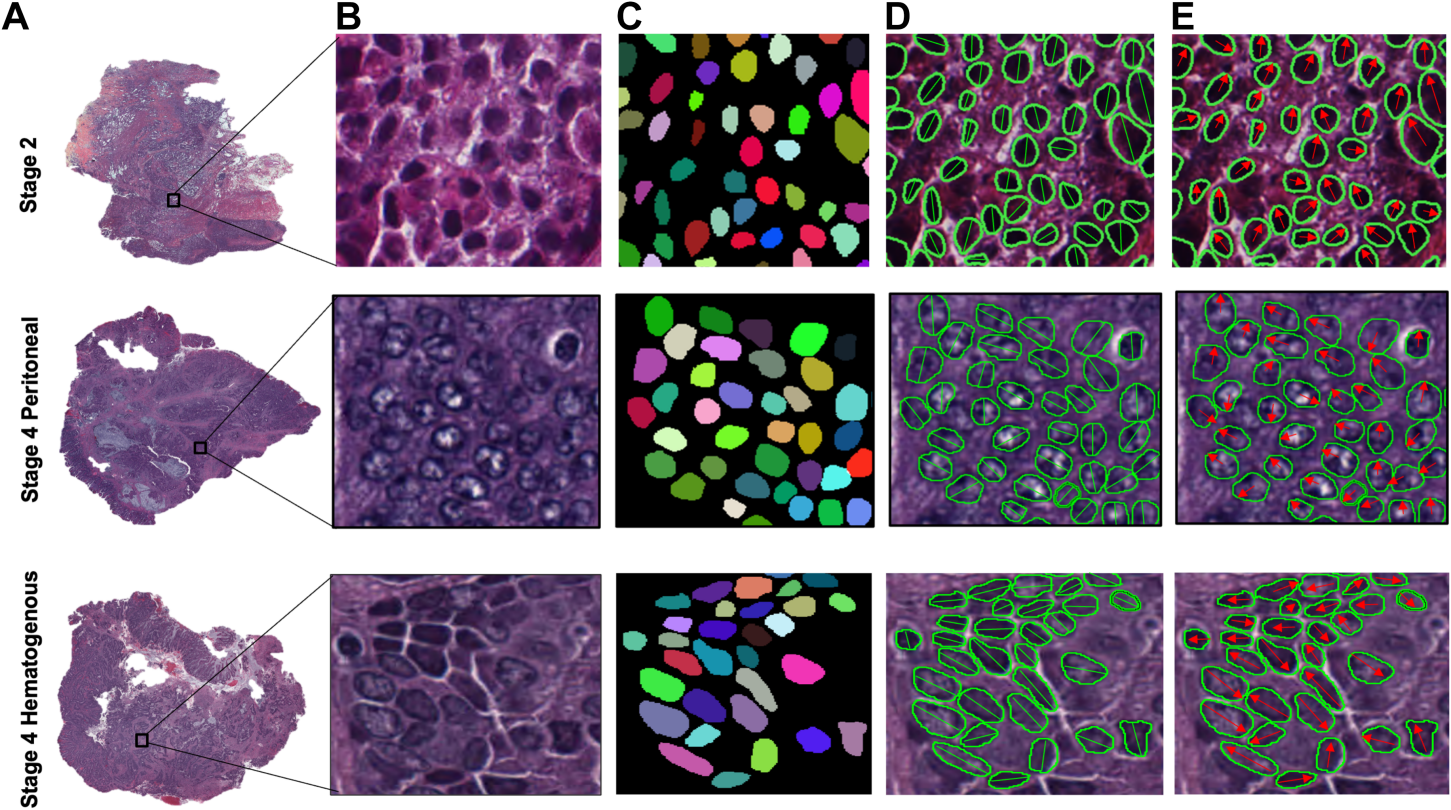
Illustration of the features extracted from the segmented tumor nuclei for stage II CC and stage IV CC with both peritoneal and hematogenous metastases. (A) Patches of size 1000 × 1000 pixels were extracted from the tumor region of the input WSI. (B) An input patch to the nuclei segmentation module. (C) Output of the nuclei segmentation module, where each nucleus is shown using different colors to show the separation of touching and overlapping nuclei. (D) Nuclear shape features quantifying attributes such as circumference, area, length of major axis, etc. and (E) nuclear orientation features quantifying the direction (in red arrows) of the major axis of each of the segmented nuclei are shown as an illustration.

Figure 3 shows an illustrative example of the quantitative nuclear features extracted to distinguish between stage II and stage IV CC. Features pertaining to nuclear shape and orientation were identified as the most important ones for distinguishing between stage II and stage IV CC. From Figure 3, we can deduce that the nuclei of stage II CC were in general smaller and had less variation in the directionality of their principal axis compared with the nuclei of stage IV CC with hematogenous spread. Further, the nuclei of stage IV CC with peritoneal metastases had an intermediate nuclear size and less variation in their orientation than the stage IV CC with hematogenous metastases.

Additional comparative strategies involving feature selection and machine classifiers are provided in supplementary material, Table S3. Additional comparisons were conducted using other nuclear features including cell run length and graph features, and the corresponding results are provided in supplementary material, Table S4 and Figure S2.

### Experiment 2: assessing the survival characteristics of the histomorphometrically determined staging groups

**Figure 4:**
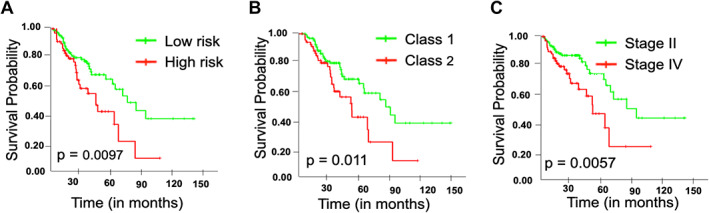
K–M curves for overall survival on the independent validation set of the TCGA‐COAD cohort. (A) The Cox proportional hazards model generated low‐risk and high‐risk categories using the top five quantitative features. (B) Classes generated using unsupervised hierarchical clustering based on the top five quantitative nuclear features. (C) Original stage labels available in the TCGA‐COAD cohort's clinical data.

Survival analysis was conducted on the independent TCGA validation set to evaluate the efficacy of the model‐generated classification labels for individual tumors to see if there were differences in the survival probabilities across the cases labeled as stage II or IV by the model. The Kaplan–Meier (KM) survival curves of overall survival for the two categories are shown in Figure 4A. The Cox proportional hazards model yielded a hazard ratio of 2.196 (95% CI 1.24–3.88) with a concordance index of 0.71 when only the top five features were used to compute the hazards for high‐risk tumors, treating low‐risk tumors as the baseline. Figure 4B shows the KM curves when unsupervised hierarchical clustering was used to categorize patients into two groups based on the top five quantitative nuclear features. The Cox proportional hazards model yielded a hazard ratio of 1.951 (95% CI 1.18–3.23) with a concordance index of 0.68 when the top five features were used to compute the hazards for the two risk groups identified by the hierarchical clustering. Finally, the KM curves generated from the true stage labels available in the TCGA data are shown in Figure 4C. From Figure 4, it is evident that the top five quantitative nuclear features were able to group the CCs accurately into two categories in both a supervised (low risk versus high risk) and an unsupervised learning framework (class 1 versus class 2).

### Experiment 3: assessing the similarity of stage IV peritoneal versus hematogenous tumors versus stage II CC in terms of nuclear histomorphometric features

**Figure 5:**
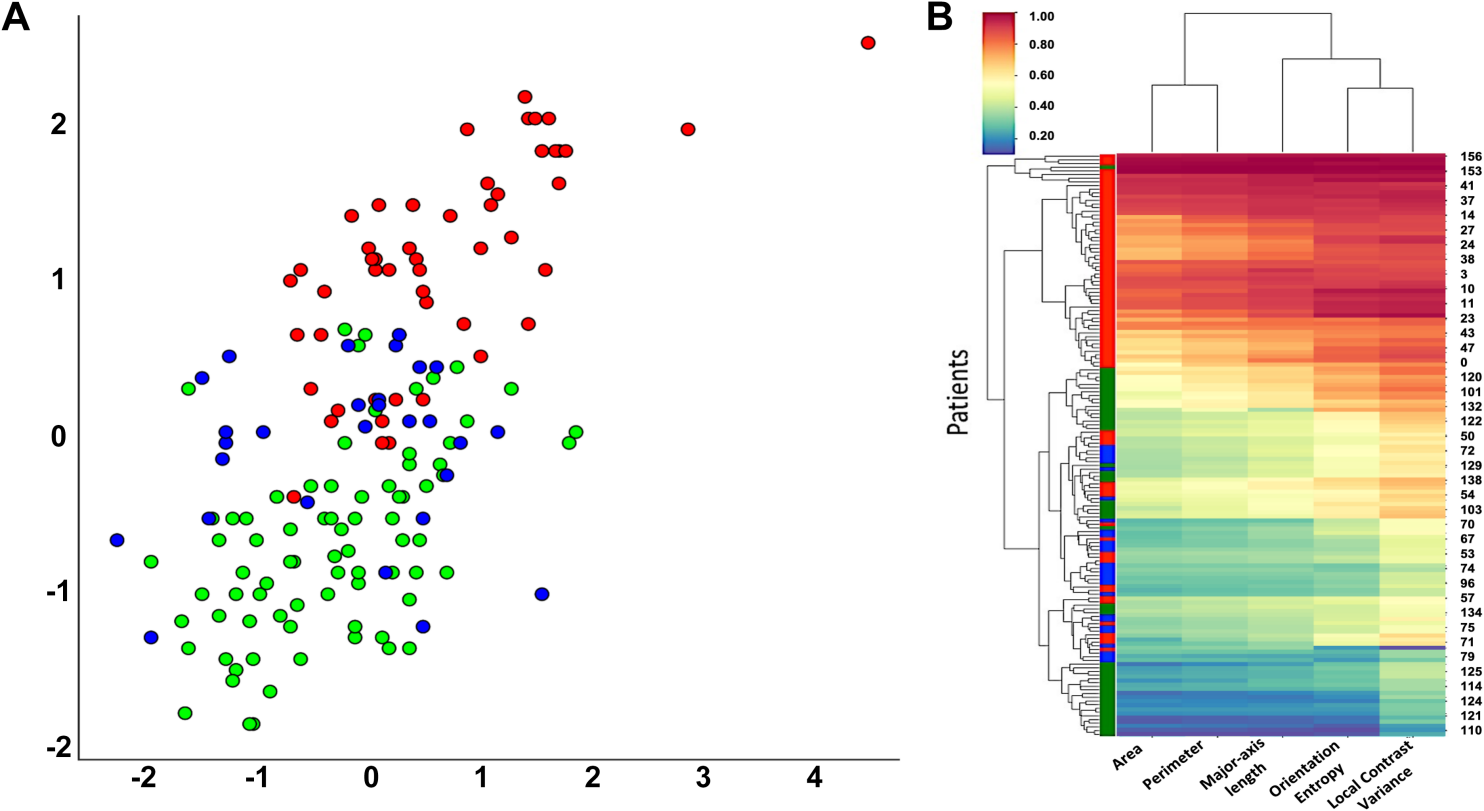
Comparison of hematogenous and peritoneal metastases of stage IV CC with stage II CC. (A) UMAP illustration of hematogenous versus peritoneal metastases of stage IV CC. (B) Unsupervised clustering‐based heatmap of the top five features that generated maximum cluster separation. True class labels are shown on the left vertical bar beside the heatmap – stage II CCs are shown in green, while blue and red represent stage IV CC with peritoneal and hematogenous metastases, respectively.

To examine the quantitative resemblance of stage IV CC with peritoneal spread to the stage II CC, we created a two‐dimensional embedding of the top five quantitative features that we extracted from each of the stage II CCs (*n* = 51) and stage IV CCs with either peritoneal (*n* = 28) or hematogenous (*n* = 79) metastases using the UMAP algorithm. This embedding is shown as a scatter plot in Figure 5A. From Figure 5A, it is clear that the stage IV CCs with peritoneal metastases adhered more closely to the stage II CCs, which did not progress, than the stage IV CCs with hematogenous spread. It should be noted, however, that stage IV CC with peritoneal spread was used neither for training nor for testing of the classifiers, and that only quantitative nuclear features were extracted from stage IV CC with peritoneal spread.

**Figure 6:**
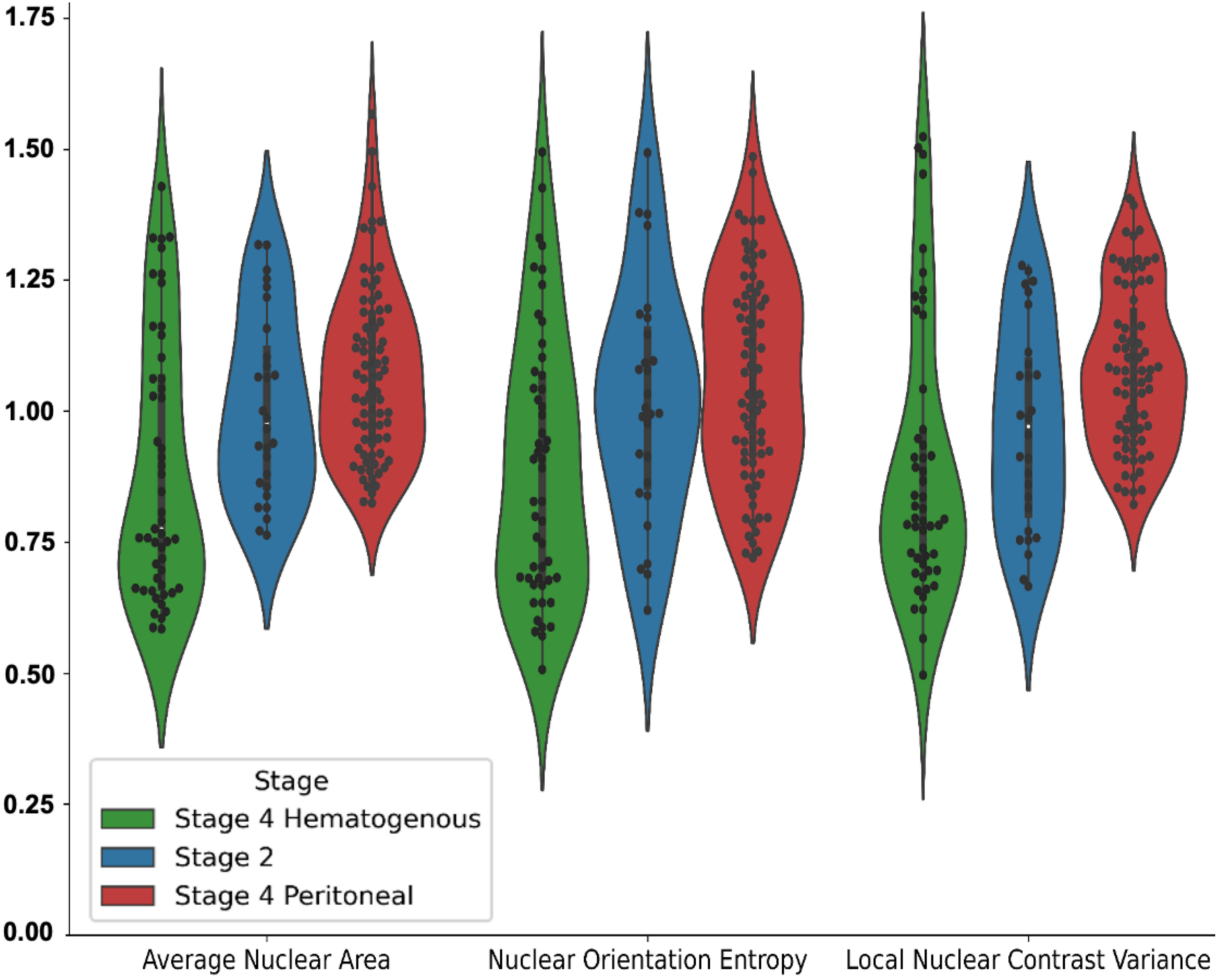
Violin plots of the top discriminatory features between stage II and stage IV CC with both peritoneal and hematogenous metastases. The best‐performing feature from each of the top feature families is shown in this illustration – average nuclear area from nuclear shape features, variance of nuclear orientation from cell‐graph tensor (CGT) features, and variance in local nuclear contrast from local cell‐cluster co‐occurrence nuclear morphology matrix (cCCM) based features.

Another way to examine the closeness of stage II CC and stage IV CC with peritoneal metastases is through unsupervised hierarchical clustering of the quantitative nuclear features. We obtained quantitative nuclear features from all validation cases and performed hierarchical clustering in the nuclear feature space. The hierarchical clustering dendogram was cut at three clusters, and the corresponding heatmap is shown in Figure 6B. Due to the large feature space (>25 000 features), the heatmap is shown only for the top five features that generated the maximum cluster separation. These features were nuclear area, nuclear perimeter, major‐axis length of nuclei, variance of nuclear contrast, and entropy of nuclear orientation – which are similar to those obtained from our previously explained supervised analysis. This indicates that these features are the most relevant features to distinguish between stage II and stage IV CC. It is also evident from Figure 5B that stage IV CCs with peritoneal metastases cluster closer to stage II CC, while stage IV CCs with hematogenous metastases form a separate cluster.

To further examine the differences between the top discriminatory features between stage II and stage IV CC, we analyzed the distributions of normalized feature values (0–1) using violin plots, as shown in Figure 6. It should be noted that the distributions of the most statistically significant features, between stage II and stage IV CC, within each of the top feature families are shown in Figure 6. From Figure 6, it is evident that the nuclei in stage IV CC were larger on average than their stage II counterparts (0.61 versus 0.26, *p* < 0.01). Additionally, the distribution of nuclear area for stage IV CC was more concentrated around the mean, with a heavy tail, compared with the nuclear area distribution of stage II CC, which was more spread out. Similar trends were observed for the other two discriminatory nuclear features. These trends indicate that the nuclei in stage IV CC were uniformly larger, while the nuclei in stage II tumors were smaller and had a higher degree of variance in their sizes. Furthermore, the entropy of the distribution of the nuclear orientation was higher for stage IV nuclei than for stage II nuclei (0.59 versus 0.40, *p* < 0.01), indicating that the nuclei in stage IV tumors have a higher degree of orientation disorder, while stage II nuclei were more uniformly oriented. Finally, stage IV nuclei show higher variance in local nuclear contrast compared with stage II nuclei (0.30 versus 0.60, *p* < 0.01), according to the features obtained from the local cell‐cluster co‐occurrence nuclear morphology matrix (cCCM; see Supplementary materials and methods for details) shown in Figure 6. Conclusively, nuclear area, perimeter, major‐axis length, nuclear contrast, and entropy of nuclear orientation were the most discriminatory features for distinguishing between stage II and stage IV colon tumors.

## Discussion

A number of studies have sought to define clinically useful prognostic markers in CC, although apart from features related to stage and grade, along with determination of microsatellite stability and BRAF status, no other markers have been universally incorporated into clinical practice [24]. The need for improved predictive markers in CC is obvious. For example, although most patients with stage II CC are cured by surgery alone, approximately 25% recur – the majority without having received post‐operative adjuvant therapy [1]. Also, 30% of all CCs present as stage III, which has an approximately 40–50% recurrence rate [50]. Even ‘low‐risk’ stage III CC (defined as having 1–3 positive lymph nodes) has a recurrence rate of at least 20%. The majority of CC recurrences are lethal [51]. Thus, a reliable CC prognostication scheme would define patients for whom intense monitoring and potential changes for management could be considered. Quantitative histomorphometric analysis (QHA) methods may play a role in better defining these patients, facilitating enhanced clinical decision support for their treating physicians.

In this study, we developed and validated a quantitative, histomorphometric‐based image risk classifier to accurately segregate stage II with at least 5‐year recurrence‐free survival from stage IV CC from digital WSIs of H&E tumor sections. We identified nuclear size features including area, perimeter, and major‐axis length along with nuclear orientation, and local variance in nuclear contrast as the top five discriminatory features that successfully distinguished between stage II and stage IV CC in a single institution validation cohort. These top five tumor nucleus features were independently validated on the TCGA‐COAD cohort cases for stage II versus stage IV classification and were also associated with patients’ overall survival outcomes for respective classes. Finally, in a unique study of the subtypes of stage IV CC, CC with dissemination into the peritoneal cavity via direct extension had nuclear features in between those of stage II CC with no evidence of recurrence and CC with hematogenous spread.

Nuclear changes are integral to cancer biology, being one of the earliest recognized features of cancer [52]. Oncogenesis, in the majority of cancer types, is associated with increasing abnormality of nuclear size and shape accompanied by chromatin and nuclear envelope irregularities. These changes are directly related to molecular events of cancer progression and are often specific for individual cancer types [53]. Routine pathological assessments of cancer nuclei are fundamental to cancer classification schemes and are incorporated into prognosis assessments in many cancers. Use of artificial intelligence for computer aided image analysis and classification has revealed important nuclear features that correlate with molecular profiles and clinical outcomes in multiple cancer types [28, 54, 55, 56, 57, 58]. Our findings support these general concepts and specifically identify quantifiable changes in nuclear features of size, entropy of orientation, and local cellular diversity, which are highly correlative with patient outcomes and are likely to be additive to currently accepted prognostic and potentially predictive markers currently used in managing CC patients.

The study appears to support the concept that cumulative molecular abnormalities, which are linked to increasing nuclear disorganization, play a pivotal role in CC patient outcomes. Previous studies have failed to identify driver gene mutation accumulation to be associated with CC metastasis [59]. However, it is now recognized that a more complex interaction of oncogenic pathways, such as activated stem‐cell programs, is associated with the likelihood of metastases – though these mechanisms need to be further elucidated [60, 61]. The CC nuclear changes, which are more prominent in stage IV than in stage II CC, are reflective of these molecular processes, although not reproducibly identifiable by a pathologist.

Peritoneal cavity seeding, as opposed to hematogenous metastases, requires different molecular events – such as tumor microenvironment interactions – though this has not been well studied. CC with direct local extension to the serosa is a known high‐risk feature of peritoneal cavity recurrence. Our data, using QHA, demonstrating that CCs with peritoneal metastases have an intermediate nuclear phenotype between CCs that do not metastasize and those with hematogenous spread is consistent with this concept.

While a number of studies have recently been reported on the use of machine learning for prognosticating survival for colon cancer from WSIs [58], our study was different from these studies in several ways. Specifically, unlike previous black‐box deep‐learning models, our approach used interpretable handcrafted features for quantitative histomorphometric analysis to develop explainable models for downstream digital pathology analysis. As demonstrated through our extensive experiments, the ‘handcrafted’ feature‐based approach showed satisfactory performance on both the initial validation set (UHCMC, S_v_) and the independent TCGA‐COAD validation set (S_t_). These results indicate that our approach is not biased to a particular dataset and could be used on external datasets without retraining, which is well‐suited to clinical adoption with further validation.

We do acknowledge that our study does have some limitations, the foremost of which being that our analysis was retrospective in nature and was performed on a limited number of patients. Furthermore, additional clinical variables (such as patient age, race, tumor grade) were not combined with image‐derived quantitative histomorphometric features for a comprehensive multivariate analysis. Future efforts will be made to investigate our model on a large patient cohort with multi‐institutional validation. Furthermore, we plan to address some of the critical questions around predictive analytics such as identifying the need for adjuvant chemotherapy for stage II CC with poor prognosis through quantitative histomorphometric analysis.

Despite the aforementioned limitations, our study demonstrated that quantitative features pertaining to nuclear area, perimeter, major‐axis length, orientation diversity, and local texture variance are useful in distinguishing between stage II CC with no long‐term metastases and stage IV CC with hematogenous spread. We validated these findings on an independent validation set obtained from the publicly available TCGA‐COAD cohort and also found an association of the identified nuclear features with patients’ survival outcomes. Finally, we also found that stage IV CCs with peritoneal carcinomatosis resemble the stage II CCs with no long‐term metastases more closely than their stage IV CC counterparts with hematogenous metastases in the selected feature space. Further studies to validate these findings on independent multi‐institutional datasets and also for potentially prospective validation are warranted.

In conclusion, this study enabled the identification of quantifiable changes in nuclear features of size, entropy of orientation, and local cellular diversity of CC WSI using QHA which are highly correlative with patient outcomes and are likely to be additive to currently accepted prognostic and potentially predictive markers used currently in managing CC patients. Furthermore, this study demonstrates the utility of artificial intelligence‐enhanced ‘handcrafted’ nuclear segmentation image analysis to accurately differentiate between CCs which do and do not metastasize. As opposed to ‘deep‐learning’ platforms, the ‘handcrafted’ approach allows for the translation of image analysis results into oncology and diagnostic pathology practice by defining the abnormalities being measured and allowing for seamless integration of other image analysis pipelines.

## Author contributions statement

NK, JW and AM were responsible for conceptualization. JW and NK curated the data. NK, RV, CC, CL and AM developed the methodology. NK and RV carried out formal analysis and validation. NK, RV, CC, CL, PF, JW and AM wrote, reviewed, and/or revised the manuscript. AM and JW administered the project, supervised the study, and acquired funding. All the authors read, and agreed with, the final version of the manuscript.

## Supporting information


Supplementary materials and methods
**Figure S1**. AUC‐ROC curves for UHCMC and TCGA validation sets for the top five most significant discriminatory features for classification between stage II and stage IV CC with hematogenous metastases using a random forest classifier
**Figure S2**. AUC‐ROC curves for CMC and TCGA validation sets for each feature using a Wilcoxon rank‐sum test for feature selection and random forest as the classification algorithm
**Figure S3**. (A) Example patch with boundaries of nuclei highlighted in green. (B) Table summarizing the first‐order statistics calculated from the nuclei within the patch (referred to in Supplementary materials and methods)
**Figure S4**. An example of a Delaunay Triangulation graph (referred to in Supplementary materials and methods)
**Figure S5**. Example of nuclear Haralick features texture features (referred to in Supplementary materials and methods)
**Figure S6**. Relationship between alpha and CCG (referred to in Supplementary materials and methods)
**Figure S7**. Nuclei orientation features obtained from cell‐graph tensors (referred to in Supplementary materials and methods)
**Figure S8**. Illustration of the basic concept of cell run‐length graph with real image examples (referred to in Supplementary materials and methods)
**Figure S9**. Flowchart for cellular diversity computation (referred to in Supplementary materials and methods)
**Table S1**. Number of whole slide images used in the training, validation, and test sets for experiments reported in this article
**Table S2**. Hyperparameter settings of our tumor and nuclei segmentation convolutional neural networks (CNNs)
**Table S3**. Classification performance of all features as a function of the statistical test of significance (for feature selection) and machine learning model (for classification)
**Table S4**. Classification performance (in terms of AUC‐ROC) of each feature family using a Wilcoxon rank‐sum test for feature selection and random forest as the classification algorithm
**Table S5**. Thirteen Haralick measurements of the co‐occurrence matrix (CM) (referred to in Supplementary materials and methods)

## Data Availability

Our tumor segmentation, nucleus segmentation, and feature extraction/classification codes are publicly available through the following GitHub links. We hope that the research community will be able to use the code to re‐implement our algorithms for their specific‐use cases. Tumor Segmentation Code: https://github.com/neerajkumarvaid/Tumor-Segmentation Nuclei Segmentation Code: https://github.com/ruchikaverma-iitg/Nuclei-Segmentation/tree/master/mrcnn Feature Extraction/Classification Code: https://github.com/DominitianusChen/DigitalPathomicExtraction.git Image identifiers of the TCGA‐COAD cases used in this study are available online at shorturl.at/apELM. All the TCGA‐COAD cohort‐associated data (WSIs, clinical and genomic information) are publicly available at the Genomics Data Commons platform (https://portal.gdc.cancer.gov/).

## References

[1] Quasar Collaborative Group , Gray R , Barnwell J , *et al*. Adjuvant chemotherapy versus observation in patients with colorectal cancer: a randomised study. Lancet 2007; 370: 2020–2029. 18083404 10.1016/S0140-6736(07)61866-2

[2] Brouwer NPM , Bos ACRK , Lemmens VEPP , *et al*. An overview of 25 years of incidence, treatment and outcome of colorectal cancer patients. Int J Cancer 2018; 143: 2758–2766. 30095162 10.1002/ijc.31785 PMC6282554

[3] Jessup JM , Goldberg RM , Asare EA , *et al*. Emerging prognostic factors for clinical care. In AJCC Cancer Staging Manual (Vol. 8, 8th edn), Amin MB , Edge SB , Greene FL , *et al*. (eds). New York: Springer, 2016.

[4] Niedzwiecki D , Bertagnolli MM , Warren RS , *et al*. Documenting the natural history of patients with resected stage II adenocarcinoma of the colon after random assignment to adjuvant treatment with edrecolomab or observation: results from CALGB 9581. J Clin Oncol 2011; 29: 3146–3152. 21747085 10.1200/JCO.2010.32.5357 PMC3157980

[5] Kuebler JP , Wieand HS , O'Connell MJ , *et al*. Oxaliplatin combined with weekly bolus fluorouracil and leucovorin as surgical adjuvant chemotherapy for stage II and III colon cancer: results from NSABP C‐07. J Clin Oncol 2007; 25: 2198–2204. 17470851 10.1200/JCO.2006.08.2974

[6] Tournigand C , André T , Bonnetain F , *et al*. Adjuvant therapy with fluorouracil and oxaliplatin in stage II and elderly patients (between ages 70 and 75 years) with colon cancer: subgroup analyses of the Multicenter International Study of Oxaliplatin, Fluorouracil, and Leucovorin in the Adjuvant Treatment of Colon Cancer trial. J Clin Oncol 2012; 30: 3353–3360. 22915656 10.1200/JCO.2012.42.5645

[7] Benson AB , Schrag D , Somerfield MR , *et al*. American Society of Clinical Oncology recommendations on adjuvant chemotherapy for stage II colon cancer. J Clin Oncol 2004; 22: 3408–3419. 15199089 10.1200/JCO.2004.05.063

[8] Figueredo A , Charette ML , Maroun J , *et al*. Adjuvant therapy for stage II colon cancer: a systematic review from the Cancer Care Ontario Program in evidence‐based care's gastrointestinal cancer disease site group. J Clin Oncol 2004; 22: 3395–3407. 15199087 10.1200/JCO.2004.03.087

[9] Gill S , Loprinzi CL , Sargent DJ , *et al*. Pooled analysis of fluorouracil‐based adjuvant therapy for stage II and III colon cancer: who benefits and by how much? J Clin Oncol 2004; 22: 1797–1806. 15067028 10.1200/JCO.2004.09.059

[10] Crispino P , de Toma G , Ciardi A , *et al*. Role of desmoplasia in recurrence of stage II colorectal cancer within five years after surgery and therapeutic implication. Cancer Invest 2008; 26: 419–425. 18443963 10.1080/07357900701788155

[11] Danielsen HE , Hveem TS , Domingo E , *et al*. Prognostic markers for colorectal cancer: estimating ploidy and stroma. Ann Oncol 2018; 29: 616–623. 29293881 10.1093/annonc/mdx794 PMC5889021

[12] Ryan É , Khaw YL , Creavin B , *et al*. Tumor budding and PDC grade are stage independent predictors of clinical outcome in mismatch repair deficient colorectal cancer. Am J Surg Pathol 2018; 42: 60–68. 29112018 10.1097/PAS.0000000000000931

[13] Lugli A , Kirsch R , Ajioka Y , *et al*. Recommendations for reporting tumor budding in colorectal cancer based on the International Tumor Budding Consensus Conference (ITBCC) 2016. Mod Pathol 2017; 30: 1299–1311. 28548122 10.1038/modpathol.2017.46

[14] Ueno H , Ishiguro M , Nakatani E , *et al*. Prospective multicenter study on the prognostic and predictive impact of tumor budding in stage II colon cancer: results from the SACURA trial. J Clin Oncol 2019; 37: 1886–1894. 31180819 10.1200/JCO.18.02059 PMC6675595

[15] Chalabi M , Fanchi LF , Dijkstra KK , *et al*. Neoadjuvant immunotherapy leads to pathological responses in MMR‐proficient and MMR‐deficient early‐stage colon cancers. Nat Med 2020; 26: 566–576. 32251400 10.1038/s41591-020-0805-8

[16] Lee H , Sha D , Foster NR , *et al*. Analysis of tumor microenvironmental features to refine prognosis by T, N risk group in patients with stage III colon cancer (NCCTG N0147) (Alliance). Ann Oncol 2020; 31: 487–494. 32165096 10.1016/j.annonc.2020.01.011 PMC7372727

[17] Compton CC , Fielding LP , Burgart LJ , *et al*. Prognostic factors in colorectal cancer. College of American Pathologists Consensus Statement 1999. Arch Pathol Lab Med 2000; 124: 979–994. 10888773 10.5858/2000-124-0979-PFICC

[18] Maoz A , Dennis M , Greenson JK . The Crohn's‐like lymphoid reaction to colorectal cancer‐tertiary lymphoid structures with immunologic and potentially therapeutic relevance in colorectal cancer. Front Immunol 2019; 10: 1884. 31507584 10.3389/fimmu.2019.01884 PMC6714555

[19] Panarelli NC , Hammer STG , Lin J , *et al*. Reproducibility of AJCC criteria for classifying deeply invasive colon cancers is suboptimal for consistent cancer staging. Am J Surg Pathol 2020; 44: 1381–1388. 32931163 10.1097/PAS.0000000000001510

[20] Klaver CEL , Bulkmans N , Drillenburg P , *et al*. Interobserver, intraobserver, and interlaboratory variability in reporting pT4a colon cancer. Virchows Arch 2020; 476: 219–230. 31616981 10.1007/s00428-019-02663-0 PMC7028812

[21] Bera K , Schalper KA , Rimm DL , *et al*. Artificial intelligence in digital pathology – new tools for diagnosis and precision oncology. Nat Rev Clin Oncol 2019; 16: 703–715. 31399699 10.1038/s41571-019-0252-y PMC6880861

[22] Madabhushi A , Feldman MD , Leo P . Deep‐learning approaches for Gleason grading of prostate biopsies. Lancet Oncol 2020; 21: 187–189. 31926804 10.1016/S1470-2045(19)30793-4

[23] Muzny DM , Bainbridge MN , Chang K , *et al*. Comprehensive molecular characterization of human colon and rectal cancer. Nature 2012; 487: 330–337. 22810696 10.1038/nature11252 PMC3401966

[24] Sepulveda AR , Hamilton SR , Allegra CJ , *et al*. Molecular biomarkers for the evaluation of colorectal cancer: guideline from the American Society for Clinical Pathology, College of American Pathologists, Association for Molecular Pathology, and the American Society of Clinical Oncology. J Clin Oncol 2017; 35: 1453–1486. 28165299 10.1200/JCO.2016.71.9807

[25] LeCun Y , Bengio Y , Hinton G . Deep learning. Nature 2015; 521: 436–444. 26017442 10.1038/nature14539

[26] Jang H‐J , Lee A , Kang J , *et al*. Prediction of clinically actionable genetic alterations from colorectal cancer histopathology images using deep learning. World J Gastroenterol 2020; 26: 6207–6223. 33177794 10.3748/wjg.v26.i40.6207 PMC7596644

[27] Sari CT , Gunduz‐Demir C . Unsupervised feature extraction via deep learning for histopathological classification of colon tissue images. IEEE Trans Med Imaging 2019; 38: 1139–1149. 30403624 10.1109/TMI.2018.2879369

[28] Echle A , Grabsch HI , Quirke P , *et al*. Clinical‐grade detection of microsatellite instability in colorectal tumors by deep learning. Gastroenterology 2020; 159: 1406–1416. 32562722 10.1053/j.gastro.2020.06.021 PMC7578071

[29] Kather JN , Pearson AT , Halama N , *et al*. Deep learning can predict microsatellite instability directly from histology in gastrointestinal cancer. Nat Med 2019; 25: 1054–1056. 31160815 10.1038/s41591-019-0462-y PMC7423299

[30] Bulten W , Pinckaers H , van Boven H , *et al*. Automated deep‐learning system for Gleason grading of prostate cancer using biopsies: a diagnostic study. Lancet Oncol 2020; 21: 233–241. 31926805 10.1016/S1470-2045(19)30739-9

[31] Ström P , Kartasalo K , Olsson H , *et al*. Artificial intelligence for diagnosis and grading of prostate cancer in biopsies: a population‐based, diagnostic study. Lancet Oncol 2020; 21: 222–232. 31926806 10.1016/S1470-2045(19)30738-7

[32] Coudray N , Ocampo PS , Sakellaropoulos T , *et al*. Classification and mutation prediction from non‐small cell lung cancer histopathology images using deep learning. Nat Med 2018; 24: 1559–1567. 30224757 10.1038/s41591-018-0177-5 PMC9847512

[33] Liao H , Long Y , Han R , *et al*. Deep learning‐based classification and mutation prediction from histopathological images of hepatocellular carcinoma. Clin Transl Med 2020; 10: e102. 32536036 10.1002/ctm2.102 PMC7403820

[34] Lu C , Bera K , Wang X , *et al*. A prognostic model for overall survival of patients with early‐stage non‐small cell lung cancer: a multicentre, retrospective study. Lancet Digit Health 2020; 2: e594–e606. 33163952 10.1016/s2589-7500(20)30225-9 PMC7646741

[35] Verma R , Kumar N , Sethi A , *et al*. Detecting multiple sub‐types of breast cancer in a single patient. *2016 IEEE International Conference on Image Processing (ICIP)*, 2016; 2648–2652.

[36] Corredor G , Wang X , Zhou Y , *et al*. Spatial architecture and arrangement of tumor‐infiltrating lymphocytes for predicting likelihood of recurrence in early‐stage non‐small cell lung cancer. Clin Cancer Res 2019; 25: 1526–1534. 30201760 10.1158/1078-0432.CCR-18-2013 PMC6397708

[37] Vamathevan J , Clark D , Czodrowski P , *et al*. Applications of machine learning in drug discovery and development. Nat Rev Drug Discov 2019; 18: 463–477. 30976107 10.1038/s41573-019-0024-5 PMC6552674

[38] Braman N , Prasanna P , Whitney J , *et al*. Association of peritumoral radiomics with tumor biology and pathologic response to preoperative targeted therapy for HER2 (ERBB2)‐positive breast cancer. JAMA Netw Open 2019; 2: e192561. 31002322 10.1001/jamanetworkopen.2019.2561 PMC6481453

[39] Kong J , Cooper LAD , Wang F , *et al*. Machine‐based morphologic analysis of glioblastoma using whole‐slide pathology images uncovers clinically relevant molecular correlates. PLoS One 2013; 8: e81049. 24236209 10.1371/journal.pone.0081049 PMC3827469

[40] Kumar N , Verma R , Arora A , *et al*. Convolutional neural networks for prostate cancer recurrence prediction. *Proc. SPIE 10140, Medical Imaging 2017: Digital Pathology*, 101400H (1 March 2017); 10.1117/12.2255774.

[41] Simon I , Pound CR , Partin AW , *et al*. Automated image analysis system for detecting boundaries of live prostate cancer cells. Cytometry 1998; 31: 287–294. 9551604 10.1002/(sici)1097-0320(19980401)31:4<287::aid-cyto8>3.0.co;2-g

[42] Basavanhally A , Ganesan S , Feldman M , *et al*. Multi‐field‐of‐view framework for distinguishing tumor grade in ER+ breast cancer from entire histopathology slides. IEEE Trans Biomed Eng 2013; 60: 2089–2099. 23392336 10.1109/TBME.2013.2245129 PMC5778451

[43] Lewis JS , Ali S , Luo J , *et al*. A quantitative histomorphometric classifier (QuHbIC) identifies aggressive versus indolent p16‐positive oropharyngeal squamous cell carcinoma. Am J Surg Pathol 2014; 38: 128–137. 24145650 10.1097/PAS.0000000000000086 PMC3865861

[44] Vatandoust S , Price TJ , Karapetis CS . Colorectal cancer: metastases to a single organ. World J Gastroenterol 2015; 21: 11767–11776. 26557001 10.3748/wjg.v21.i41.11767 PMC4631975

[45] Kumar N , Verma R , Anand D , *et al*. A multi‐organ nucleus segmentation challenge. IEEE Trans Med Imaging 2020; 39: 1380–1391. 31647422 10.1109/TMI.2019.2947628 PMC10439521

[46] Simonyan K , Zisserman A . Very deep convolutional networks for large‐scale image recognition. *arXiv*: 1409.1556 [cs]. 2015. Available from: http://arxiv.org/abs/1409.1556 [Not peer reviewed].

[47] Vahadane A , Peng T , Sethi A , *et al*. Structure‐preserving color normalization and sparse stain separation for histological images. IEEE Trans Med Imaging 2016; 35: 1962–1971. 27164577 10.1109/TMI.2016.2529665

[48] He K , Gkioxari G , Dollár P , *et al*. Mask R‐CNN. *arXiv*: 1703.06870 [cs]. 2017. Available from: http://arxiv.org/abs/1703.06870 [Not peer reviewed].

[49] Lu C , Koyuncu C , Corredor G , *et al*. Feature‐driven local cell graph (FLocK): new computational pathology‐based descriptors for prognosis of lung cancer and HPV status of oropharyngeal cancers. Med Image Anal 2021; 68: 101903. 33352373 10.1016/j.media.2020.101903 PMC7855877

[50] Moertel CG , Fleming TR , Macdonald JS , *et al*. Levamisole and fluorouracil for adjuvant therapy of resected colon carcinoma. N Engl J Med 1990; 322: 352–358. 2300087 10.1056/NEJM199002083220602

[51] Douillard J‐Y , Oliner KS , Siena S , *et al*. Panitumumab–FOLFOX4 treatment and *RAS* mutations in colorectal cancer. N Engl J Med 2013; 369: 1023–1034. 24024839 10.1056/NEJMoa1305275

[52] Lebert H (1813–1878). *Physiologie Pathologique, ou Recherches chimiques, expérimentales et microscopiques sur l'inflammation, la tuberculisation, les tumeurs, la formation du cal, etc*., Tome 1. JB Baillière: Paris, 1845. Available from: https://gallica.bnf.fr/ark:/12148/bpt6k61086282.

[53] Zink D , Fischer AH , Nickerson JA . Nuclear structure in cancer cells. Nat Rev Cancer 2004; 4: 677–687. 15343274 10.1038/nrc1430

[54] Whitney J , Corredor G , Janowczyk A , *et al*. Quantitative nuclear histomorphometry predicts oncotype DX risk categories for early stage ER+ breast cancer. BMC Cancer 2018; 18: 610. 29848291 10.1186/s12885-018-4448-9 PMC5977541

[55] Romo‐Bucheli D , Janowczyk A , Gilmore H , *et al*. Automated tubule nuclei quantification and correlation with oncotype DX risk categories in ER+ breast cancer whole slide images. Sci Rep 2016; 6: 32706. 27599752 10.1038/srep32706 PMC5013328

[56] Chandramouli S , Leo P , Lee G , *et al*. Computer extracted features from initial H&E tissue biopsies predict disease progression for prostate cancer patients on active surveillance. Cancers 2020; 12: E2708. 32967377 10.3390/cancers12092708 PMC7563653

[57] Lu C , Lewis JS , Dupont WD , *et al*. An oral cavity squamous cell carcinoma quantitative histomorphometric‐based image classifier of nuclear morphology can risk stratify patients for disease‐specific survival. Mod Pathol 2017; 30: 1655–1665. 28776575 10.1038/modpathol.2017.98 PMC6128166

[58] Skrede OJ , de Raedt S , Kleppe A , *et al*. Deep learning for prediction of colorectal cancer outcome: a discovery and validation study. Lancet 2020; 395: 350–360. 32007170 10.1016/S0140-6736(19)32998-8

[59] Jones S , Chen W‐D , Parmigiani G , *et al*. Comparative lesion sequencing provides insights into tumor evolution. Proc Natl Acad Sci U S A 2008; 105: 4283–4288. 18337506 10.1073/pnas.0712345105 PMC2393770

[60] Patel SA , Rodrigues P , Wesolowski L , *et al*. Genomic control of metastasis. Br J Cancer 2021; 124: 3–12. 33144692 10.1038/s41416-020-01127-6 PMC7782491

[61] Waylen LN , Nim HT , Martelotto LG , *et al*. From whole‐mount to single‐cell spatial assessment of gene expression in 3D. Commun Biol 2020; 3: 1–11. 33097816 10.1038/s42003-020-01341-1 PMC7584572

[62] Delaunay B . Sur la sphere vide. A la memoire de Georges Voronoi. Bull Acad Sci USSR 1934; 6: 793–800.

[63] Sharma H , Zerbe N , Lohmann S , *et al*. A review of graph‐based methods for image analysis in digital histopathology. Diagn Pathol 2015. Available from: http://www.diagnosticpathology.eu/content/index.php/dpath/article/view/61.

[64] Nanni L , Brahnam S , Ghidoni S , *et al*. Different approaches for extracting information from the co‐occurrence matrix. PLoS One 2013; 8: e83554. 24386228 10.1371/journal.pone.0083554 PMC3873395

[65] Haralick RM , Shanmugam K , Dinstein I . Textural features for image classification. IEEE Trans Syst Man Cybern 1973; SMC‐3: 610–621.

[66] Lu C , Wang X , Prasanna P , *et al*. Feature driven local cell graph (FeDeG): predicting overall survival in early stage lung cancer. In Medical Image Computing and Computer Assisted Intervention – MICCAI 2018, Frangi AF , Schnabel JA , Davatzikos C , *et al*. (eds). Springer International Publishing: Cham, 2018; 407–416.

[67] Glorot X , Bengio Y . Understanding the difficulty of training deep feedforward neural networks. In: *Proceedings of the Thirteenth International Conference on Artificial Intelligence and Statistics*. JMLR Workshop and Conference Proceedings, 2010; 249–256. Available from: https://proceedings.mlr.press/v9/glorot10a.html

