# Supplementary material for: Computer‐extracted features of nuclear morphology in hematoxylin and eosin images distinguish stage II and IV colon tumors
Source: J Pathol. 2022 Feb 22;257(1):17–28. doi: 10.1002/path.5864 (PMC9007877; doi:10.1002/path.5864)
Supplement: Supplementary file 1 — Supplementary materials and methods Figure S1. AUC‐ROC curves for UHCMC and TCGA validation sets for the top five most significant discriminatory features for classification between stage II and stage IV CC with hematogenous metastases using a random forest classifier Figure S2. AUC‐ROC curves for CMC and TCGA validation sets for each feature using a Wilcoxon rank‐sum test for feature selection and random forest as the classification algorithm Figure S3. (A) Example patch with boundaries of nuclei highlighted in green. (B) Table summarizing the first‐order statistics calculated from the nuclei within the patch (referred to in Supplementary materials and methods) Figure S4. An example of a Delaunay Triangulation graph (referred to in Supplementary materials and methods) Figure S5. Example of nuclear Haralick features texture features (referred to in Supplementary materials and methods) Figure S6. Relationship between alpha and CCG (referred to in Supplementary materials and methods) Figure S7. Nuclei orientation features obtained from cell‐graph tensors (referred to in Supplementary materials and methods) Figure S8. Illustration of the basic concept of cell run‐length graph with real image examples (referred to in Supplementary materials and methods) Figure S9. Flowchart for cellular diversity computation (referred to in Supplementary materials and methods) Table S1. Number of whole slide images used in the training, validation, and test sets for experiments reported in this article Table S2. Hyperparameter settings of our tumor and nuclei segmentation convolutional neural networks (CNNs) Table S3. Classification performance of all features as a function of the statistical test of significance (for feature selection) and machine learning model (for classification) Table S4. Classification performance (in terms of AUC‐ROC) of each feature family using a Wilcoxon rank‐sum test for feature selection and random forest as the classification algorithm Table S5. Thirteen Ha [file PATH-257-17-s001.pdf]

Computer-extracted features of nuclear morphology in hematoxylin and eosin images distinguish stage II and IV colon tumors

N Kumar *et al. J Pathol* DOI: 10.1002/path.5864

Supplementary materials and methods

Supplementary Figures S1–S9

Supplementary Tables S1–S5

Supplementary materials and methods

Reference numbers refer to the reference list in the main text

Segmentation of nuclei

Mask R-CNN [48] was used as the model for nucleus segmentation that gave two binary masks as its output – one showed the probability of each pixel belonging inside or outside the nuclei and the other showed the probability of a pixel being the boundary pixel of the segmented nuclei [45]. A combination of the binary cross entropy loss function (for inside versus outside nuclei) and hinge loss function (for boundary versus non-boundary) was employed during the training phase of the network. An illustration of the nucleus segmentation neural network is shown in the bottom row of Figure 2. The nucleus segmentation model was trained using Keras with a TensorFlow backend on a Titan X GPU running CUDA 9.0 using 1000 × 1000 crops obtained from the WSI. Supplementary material, Table S2 provides the hyperparameter setting of our nucleus segmentation Mask R-CNN [48].

The training and validation data for nucleus segmentation were obtained from an international competition on multi-organ nucleus segmentation (MoNuSeg), which released

manual annotations of around 20 000 nuclei in the training set and 10 000 nucleus annotations in the validation set and included WSI (at 40×) for seven organs obtained from 18 different hospitals to develop generalized nucleus segmentation algorithms [45]. The nucleus segmentation module used in the current project obtained an average aggregated Jaccard index (AJI) of 0.70 on the challenge's validation dataset, which is on par with the winning entry of the challenge [45].

Comparison of other feature families

Following feature extraction, all quantitative features obtained from the images in the UHCMC training set (100 stage II and 100 stage IV CCs) were used in the training phase to identify the most important features to distinguish between stage II and stage IV CC using a combination of feature selection algorithms (mRMR and WRST) and machine learning classifiers (LDA, QDA, SVM, and random forests). Supplementary material, Table S3 shows that the combination of Wilcoxon rank-sum test (WRST) for feature selection and random forest classifier for classification using the selected features gives the best class separation, with an AUC of 0.81 on the UHCMC initial validation set.

After choosing the best-performing feature selection (Wilcoxon rank-sum test) and machine learning classifier (random forest), we analyzed the ability of each feature family to separately identify which features were the most relevant for stage II versus stage IV classification for independent validation of the UHCMC and TCGA validation sets. Supplementary material, Table S4 shows that features pertaining to the nuclear size (area, perimeter, and major-axis length), entropy of the nuclear orientation, and local cellular diversity (variance of nuclear contrast) were the most important ones to distinguish between stage II and stage IV CC. The AUC-ROC curves for each feature family are

shown in supplementary material, Figure S2. It is clear from supplementary material, Table S4 and Figure S2 that nuclear shape features, orientation, and the local cellular diversity yielded the best AUC-ROC for independent validation on both the UHCMC and the TCGA validation sets.

Feature descriptions

1 – Basic shape/intensity/morphology features of nuclei: This family quantifies the first-order statistics, including mean, median, standard derivation, kurtosis, range, and skewness of all nuclear shape, size, texture, or morphology within the image. For each patch, the pre-segmented nucleus area was estimated. After that, six first-order statistics, including mean, median, standard derivation, kurtosis, range, and skewness, were calculated using all nuclei data within the patch. An example is shown in supplementary material, Figure S3. The numbers represent the pixel intensity within each nucleus. The first-order statistics could thus be calculated and are shown in supplementary material, Figure S3B.

2 – Global architecture of nuclear distribution: This family captures the nuclei arrangement and distribution in an image. Different types of global graph structures (Delaunay triangulation [62], Voronoi diagram, minimum spanning tree [63]) were constructed by using locations of nuclei as vertices. The traditional graph measurements were then calculated. An example of a Delaunay triangulation graph is shown in supplementary material, Figure S4.

3 – Nuclear Haralick texture features: All of the pixels from each nucleus were used to construct the co-occurrence matrix [64], followed by Haralick measurement extraction [65]. The 13 Haralick measurements are shown in supplementary material, Table S5. For one image, we have many nuclei and the final signature for that image is represented by the statistics of the Haralick features of all nuclei. An example of the extracted nuclear Haralick texture features is shown in supplementary material, Figure S5. The H&E image was first converted to a grayscale image. Then the gray-level co-occurrence matrix (GLCM) was calculated using the pixels from each pre-segmented nucleus. The entropy of the GLCM was then calculated using the methods introduced in supplementary material, Table S5.

4 – Local cell cluster graph (CCG): This feature family captures the nuclear architecture in the local region by constructing a local cell graph. The cell graphs were form based on the proximity of nuclei. Grouping nuclei into local clusters will construct the local nuclear graph (LNG). With the nuclei clustered, interactions between nuclei could be better characterized, and the nuclear properties which could quantify tumor morphology could be extracted more efficiently.

To construct the LNG, a set of *n* segmented nuclei (denoted as *N*), where $N = \{\eta_i, p \in \{1, 2, \ldots, n\}\}$, was used to create a graph $G = (N_G, E_G)$, where N_G represents the vertices of the graph (essentially the nuclei centroids) and E_G represents the set of edges connecting the nuclei within *G*. Construction of the LNG can be achieved by linking nearby nuclei based on vicinity criteria as follows:

$$P(\eta_u, \eta_v) = d(\eta_u, \eta_v)^{-\alpha},$$

where η_u and η_v are two vertices/nucleus in the LNG, $d(\eta_u, \eta_v)$ represents the Euclidean distance between the two nuclei, and parameter *α* was set to control the density of the graph. Intuitively, $P(\eta_u, \eta_v)$ was defined as the probability of two nuclei being connected in a graph. Thus, this probability could also quantify the possibility one of these nuclei being grown from the other. Since this probability decreases with an increase in distance, we probabilistically define an edge set *E*, such that

$$E = \{(\eta_u, \eta_v) : r < d(\eta_u, \eta_v)^{-\alpha}, \forall \eta_u, \eta_v \in N\},$$

where $r \in [0,1]$ is a parameter empirically set to be 0.2. With an exponent of $-\alpha$ for $\alpha \geq 0$, a larger value of *α* produces sparser graphs. As *α* approaches 0, the graphs become densely connected and approach a complete graph. To extract the features from different sizes of the CCG, the algorithm would iterate the parameter *α* from 0.38 to 0.52 with a step of 0.2. An example is shown in supplementary material, Figure S6 to demonstrate the CCG with different *α*.

<u>5 – Basic shape/intensity features of nuclei in the local region:</u> This family quantifies the first-order statistics, including mean, median, standard derivation, kurtosis, range, and

skewness of the basic shape/size/texture properties for nuclei in the local region defined by the local cell cluster graph (CCG).

6 – Local cell graph tensor (CGT): This feature family captures nuclear orientation's entropy in the local region defined by the CCG. The steps to generate a CCG have been described above. For a nucleus within the local graph, η_i, the nuclear orientation was calculated by first performing principal component analysis on the set of nuclei boundary points $[x_p, y_p]$ to obtain the principal components $A = [a_1, a_2]$, where a_1 describes the orientation of the cell. An angle, $\theta(\eta_i) \in [0^{\circ}, 180^{\circ}]$, was then calculated using the first principal component a_1. The orientation of cell η_i was then set to be $\theta(\eta_i)$ counterclockwise from the vector <1,0>. One example is shown in supplementary material, Figure S7.

In order to compute the co-occurring matrix, the nuclear orientation angle $\theta(\eta_i)$ was discretized, such that $\overline{\theta}(\eta_i) = \omega * \left\lfloor \frac{\theta(\eta_i)}{\omega} \right\rfloor$. In LNG, $G = (V, E)$, for each nucleus, $\eta_i \in V$, a neighborhood N_i could be defined to include all $\eta_i \in V$ where a path between η_i and η_j exists in the graph. Thus, for each neighborhood N_p, and $b = \frac{180}{\omega}$, we have a *b* by *b* co-occurrence matrix, $\mathcal{C}_{N_p}$:

$$\mathcal{C}_{N_p}(\overline{\theta}_1, \overline{\theta}_2)_{\eta_i, \eta_j \in N_p} = \sum_{\substack{\theta_1, \theta_2 = 1 \\ \theta_1 \neq \theta_2}}^{b} \begin{cases} 1, & \text{if } \theta(\eta_i) = \theta_1 \text{ and } \theta(\eta_j) = \theta_2 \\ 0, & \text{otherwise} \end{cases}$$

$\mathcal{C}_{N_i}$ could be aggregated to obtain

$$\mathcal{C}(\overline{\theta}_1, \overline{\theta}_2) = \frac{1}{n} \sum_{i=1}^{n} \mathcal{C}_{N_p}(\overline{\theta}_1, \overline{\theta}_2)$$

The entropy was then calculated using the following equation:

$$\text{Entropy} = \sum_{\overline{\theta}_1,\overline{\theta}_2} -\mathcal{C}(\overline{\theta}_1,\overline{\theta}_2)\log\mathcal{C}(\overline{\theta}_1,\overline{\theta}_2)$$

7 – Cell run length features (CRL): This feature family calculates the local cell graph's complexity across the image. An example is shown below. In supplementary material, Figure S8(b), the length of runs was numbered from 2 to 6, so that the run-length vector was R = [0,0,0,0,1]. Another example of a local cell graph is shown in supplementary material, Figure S8(c), in which the cell graph also consists of six cells. However, it contains one 5-run and one 6-run; the corresponding run-length vector was R = [0,0,0,1,1]. In terms of the total number of cell runs, the cell graph shown in supplementary material, Figure S8(c) is more complex than the one shown in supplementary material, Figure S8(b).

8 – Feature-driven local cell graph (FeDeG): FeDeG investigates the interaction between different graphs [66]. It takes nuclear morphologic features such as nuclear mean intensity into consideration during the construction of cell graphs. To calculate FeDeG features, six nuclear morphologic features that describe the nuclear shape, size, and texture were first calculated from the pre-segmented nuclei.

Next, mean-shift clustering was applied to construct the sub-graphs. For a nucleus, $Nu_n(n \in [1, N])$, a corresponding feature vector exists in the *d*-dimensional Euclidean space R^d. Thus, for $x_n \in R^d$, we had a nuclear feature vector set $X = x_1, \cdots, x_N$. For each x_n, a corresponding mode y_i, where y_i was initialized to be equal to x_n. Then y_i^u was reclusively updated based on the mean-shift vector $m_G(y_i^u)$:

$$y_i^{u+1} = y_i^u + m_G(y_i^u), 1 \leq i \leq n$$

$m_G(y_i^u)$ is the difference between the weighted mean and the center of the kernel. At the end of iteration, each x_n would find a mode y_i, which was used to generate the FeDeG. After that, a *Q*-dimensional feature space which includes the centroid location of nuclei in the image and *Q* − 2 of the nuclear morphologic features were created. Thus, a multivariate kernel was defined as follows:

$$K_{h_s,h_m}(x_i) = \frac{C}{h_s^2 h_m^{Q-2}} k\left(\left\|\frac{x_{i,s}}{h_s}\right\|\right) k(\left\|\frac{x_{i,m}}{h_m}\right\|)$$

where *k* is the profile of a radially symmetric kernel; h_s and h_m are the spatial and the nuclear morphologic component, respectively; *C* is the normalization constant; and h_s and h_m are the kernel bandwidths. A higher bandwidth would result in more neighboring data points being used to estimate the density in the Q-D feature space.

Based on the FeDeG created, four groups of features were derived to measure the interaction between FeDeGs, the size of the FeDeG, the intrinsic nuclear morphologic variation within each FeDeG, and the spatial arrangement of FeDeG.

<u>9 – Local cellular diversity features</u>: To quantify the morphology of nuclei, 11 nuclear morphologic features were calculated (listed in supplementary material, Table S5) using the segmented nuclei mask. For each nucleus η_i, we have a set of nuclear features, $M = \{m_j(\eta_i), j \in \{1 \ldots 11\}\}$. After that, an LNG was constructed using the methods described above. The co-occurrence nuclear morphology matrix (CM) was constructed for each LNG to quantify the local cellular diversity between nuclear sub-groups. If all the nuclei were identical in appearance, the co-occurrence matrix was a 1 × 1 matrix. On the other hand, the greater the diversity and range of features, the larger the co-occurrence matrix. Each

element of the co-occurrence matrices records the frequency of a co-occurring nuclear sub-group.

In order to compute the co-occurrence matrix, the nuclear morphological features $m_j(\eta_i)$ were discretized along each feature dimension, such that $\overline{m_j}(\eta_i) = \omega * \left\lfloor \frac{m_j(\eta_i)}{\omega} \right\rfloor$, where ω is a quantifying factor that divides nuclei into ω sub-classes. For instance, with ω=3, nuclear size features could be categorized into three sub-classes: nuclei with large, medium, and small size.

For an image with a total number of q LNG graphs, $G_k, k \in \{1 \ldots q\}$, all nuclei within each graph would be used to generate a corresponding CM in conjunction with a particular nuclear feature. A set of 120 basic shape features was used in this research. The constructed CM had a shape of ω by ω and was denoted as $C_{G_k}^{m_j}(a,b)$, where

$$C_{G_k}^{m_j}(a,b) = \sum_{\eta_u,\eta_v}^{G_k} \begin{cases} 1 & , \quad \text{if } m_j(\eta_u) = a \text{ and } m_j(\eta_v) = b \\ 0 & , \quad \text{otherwise} \end{cases}$$

$C_{G_k}^{m_j}(a,b)$ could in turn be expressed as follows:

$$C_{G_k}^{m_j} = \frac{1}{\sum_{a,b=1}^{\omega} C_{G_k}^{m_j}(a,b)} \begin{bmatrix} C_{G_k}^{m_j}(1,1) & C_{G_k}^{m_j}(1,2) & \cdots & C_{G_k}^{m_j}(1,\omega) \\ \vdots & \vdots & \vdots & \vdots \\ C_{G_k}^{m_j}(\omega,1) & C_{G_k}^{m_j}(\omega,2) & \cdots & C_{G_k}^{m_j}(\omega,\omega) \end{bmatrix}$$

After the CM was constructed, higher-order statistics (Haralick measurements) were extracted from each LNG. Five common statistics, including median, standard derivation, range, kurtosis, and skewness, were calculated using the Haralick measurements of each

LNG. The flowchart of extracting local cellular diversity features is shown in supplementary material, Figure S9.

Supplementary Figures S1–S9

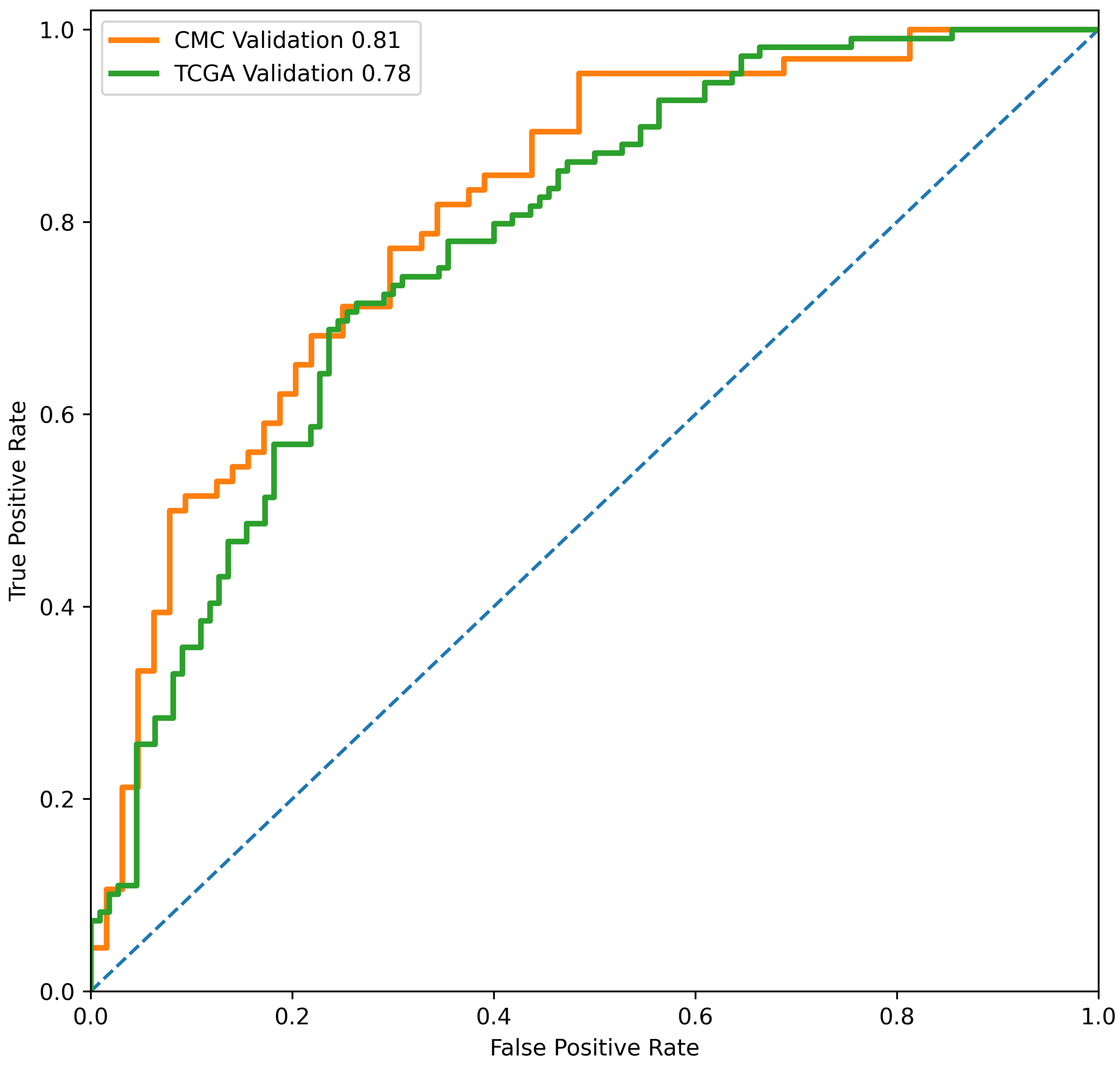

Figure S1. AUC-ROC curves for UHCMC and TCGA validation sets for the top five most significant discriminatory features for classification between stage II and stage IV CC with hematogenous metastases using a random forest classifier.

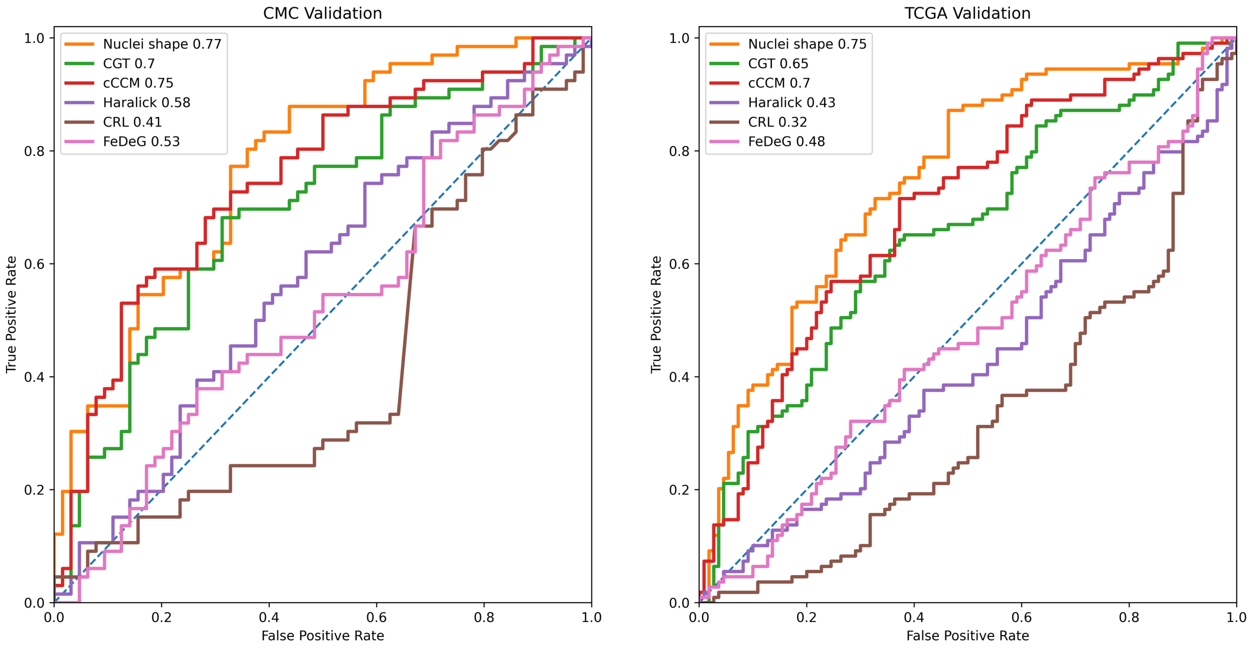

Figure S2. AUC-ROC curves for CMC and TCGA validation sets for each feature using a Wilcoxon rank-sum test for feature selection and random forest as the classification algorithm.

A.

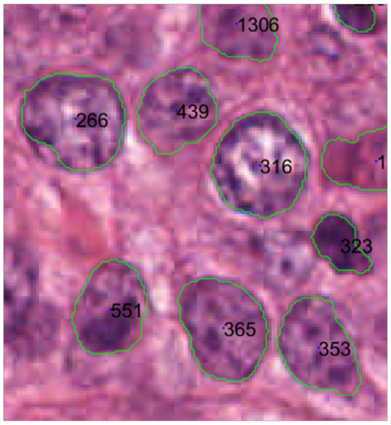

B.

Statistic	Value
Mean	489.8750
Std	341.1981
Median	359
Kurtosis	5.4024
Range	1040
Skewness	1.9918

Figure S3. (A) Example patch with boundaries of nuclei highlighted in green. (B) Table summarizing the first-order statistics calculated from the nuclei within the patch.

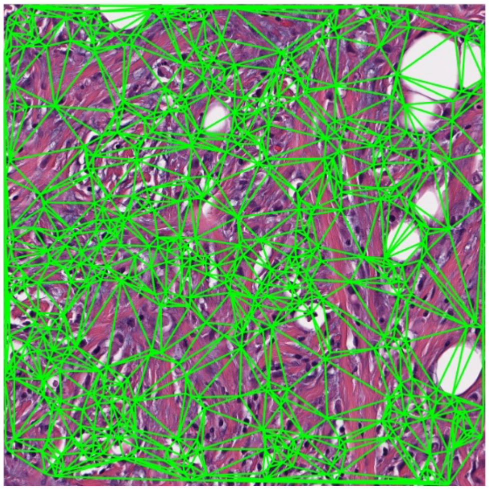

Figure S4. An example of a Delaunay triangulation graph.

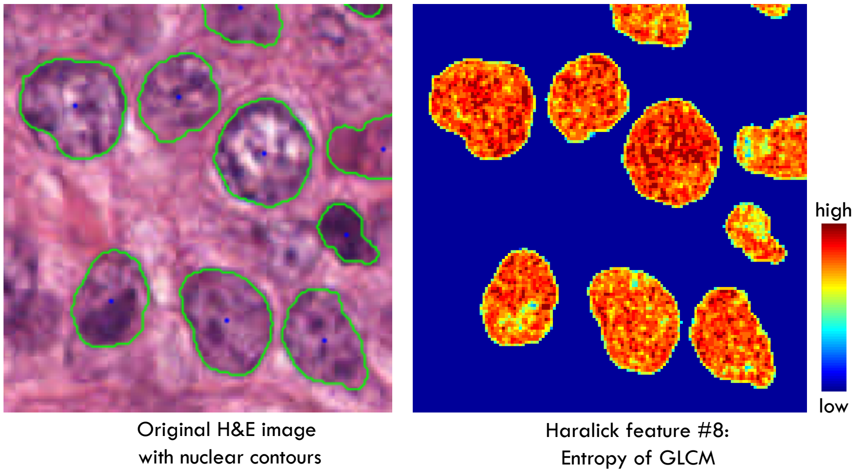

Figure S5. Example of nuclear Haralick features texture features. The original H&E patch with nuclei highlighted in green is shown on the left. The figure on the right shows the heatmap of the entropy of the gray-level co-occurrence matrix (GLCM) within the pixels of each nucleus.

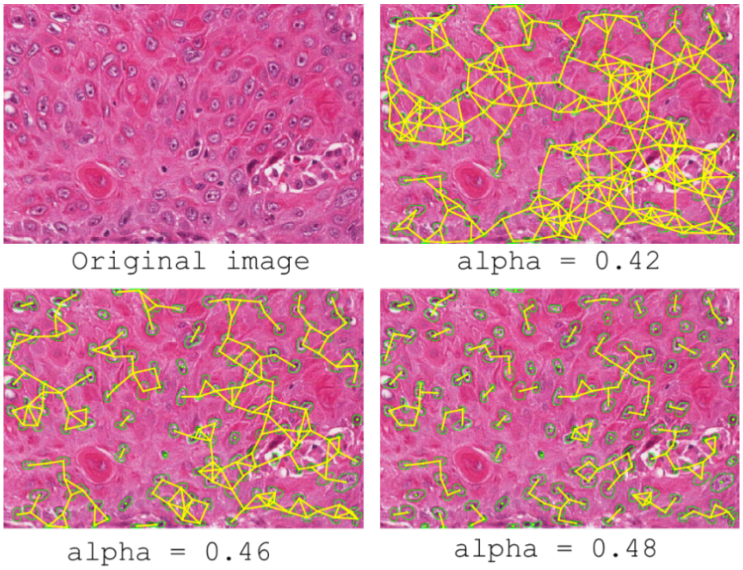

Figure S6. Relationship between alpha and CCG. The original image is shown at the top left. With an increase in alpha, the resulting CCG becomes more sparsely connected.

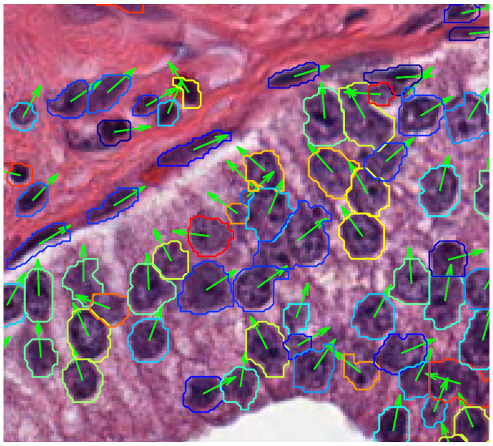

Figure S7. Nuclei orientation features obtained from cell-graph tensors.

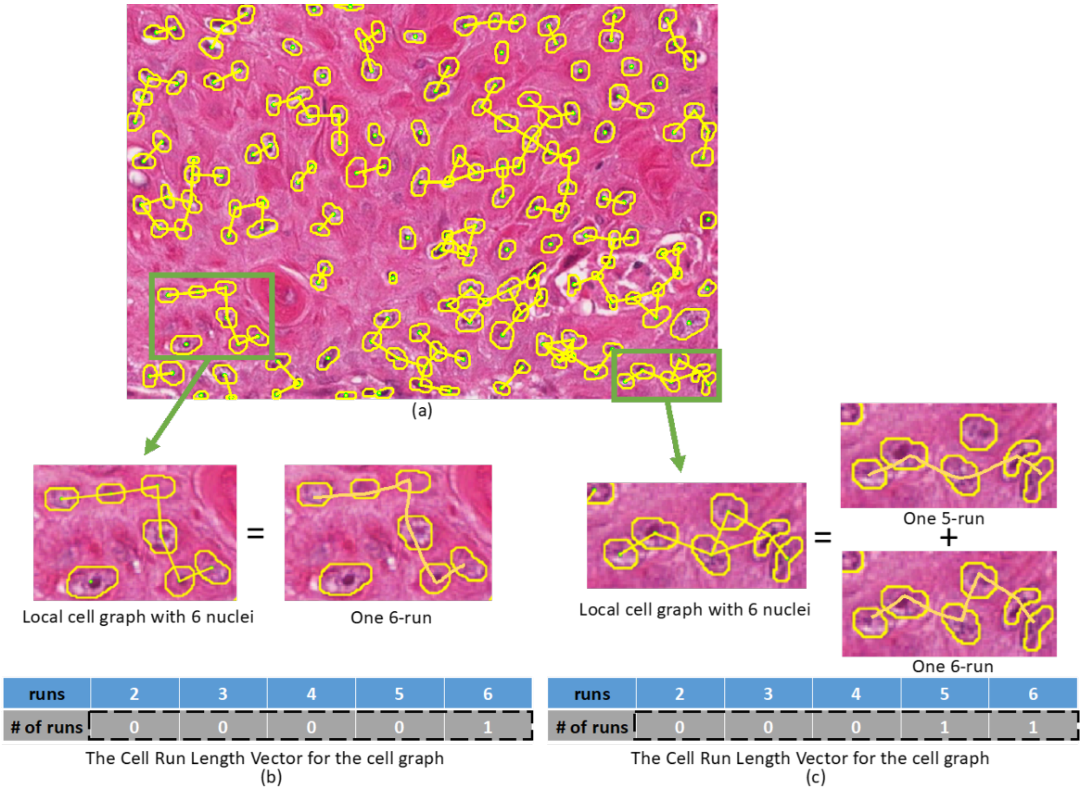

Figure S8. Illustration of the basic concept of cell run-length graph with real image examples. (a) The original H&E-stained image with pre-segmented nuclear contours and corresponding local cell graphs. Two typical local cell graphs (both 6-cell cliques) are shown in b and c, respectively. In c, the cell graph is decomposed into separate cliques comprising one 5-run and one 6-run.

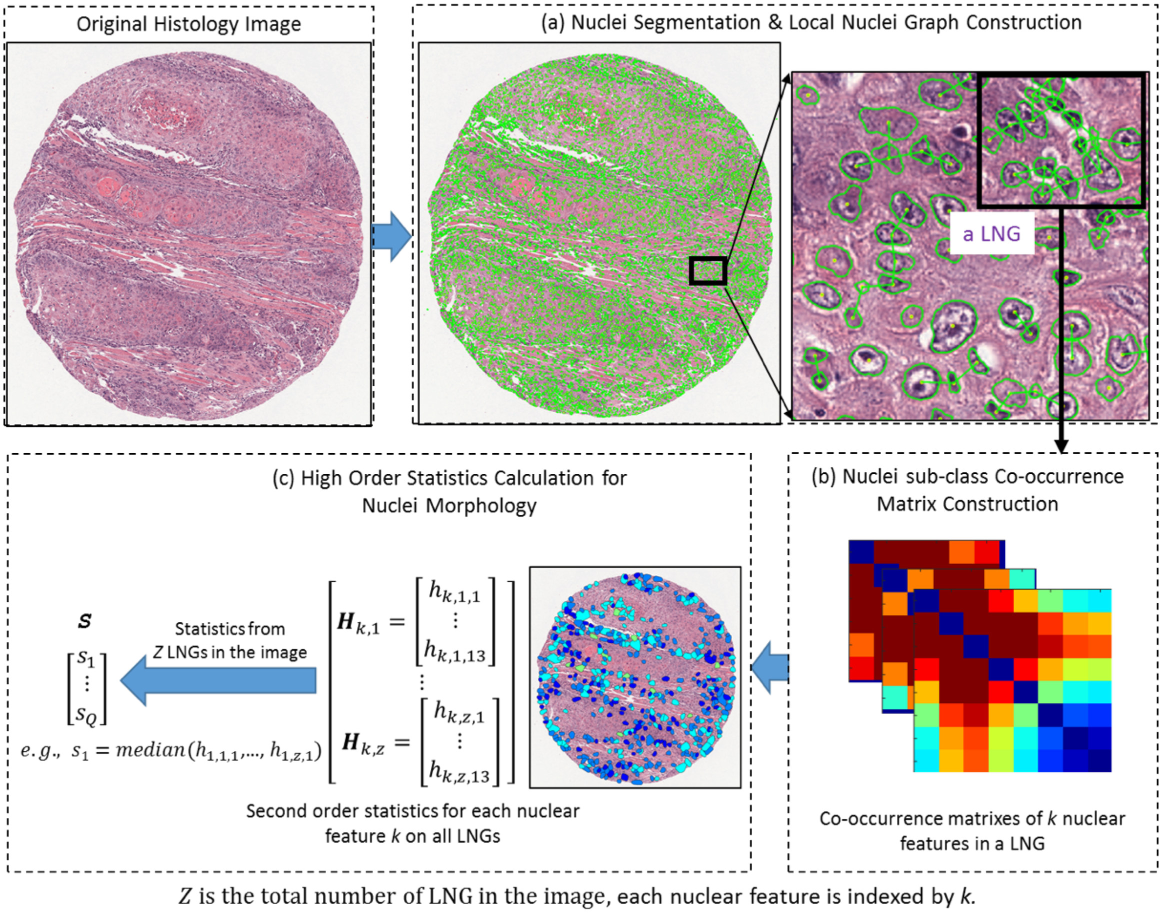

Figure S9. Flowchart for cellular diversity computation. (a) Green contours/lines indicate the nuclear boundaries and the adjacent panel shows the local nuclei graphs (LNGs) with edges between proximally located nuclei. (b) Shape, size, and texture features were then extracted for each of the nodes in the LNG and co-occurrence matrices were constructed. From these co-occurrence matrices, second-order statistics such as entropy were extracted and used to construct the cellular diversity feature vector ***S***.

Supplementary Tables S1–S5

Table S1. Number of whole slide images used in the training, validation, and test sets for experiments reported in this paper

**Dataset type**	**Stage II**	**Stage IV hematogenous**	**Stage IV peritoneal**	**Total WSIs**
UHCMC training set (S_{tr})	100	100	-	200
UHCMC validation set (S_v)	51	79	28	158
TCGA test set (S_t)	143	54	-	197
**Total WSIs**	294	233	28	555

Table S2. Hyperparameter settings of our tumor and nucleus segmentation convolutional neural networks (CNNs). Other hyperparameters including the number of convolutional layers, drop-out, skip connections, etc. were directly obtained from the original papers on VGG-18 (for tumor segmentation) [46] and Masked R-CNN (for nucleus segmentation) [48]

**Hyperparameter**	**Value for tumor segmentation VGG-18 CNN** [46]	**Value for nucleus segmentation Mask R-CNN** [48]
Input patch size	500 x 500	1000 x 1000
Weight initialization	Glorot initialization [67]	
Initial learning rate	0.01	
Optimizers	Adam	
Number of epochs	50	120
Batch size	256	128

Table S3. Classification performance of all features as a function of the statistical test of significance (for feature selection) and machine learning model (for classification)

Area under the receiver operating characteristics (UHCMC validation, S_v): LDA: linear discriminant analysis; QDA: quadratic discriminant analysis; SVM: support vector machines; mRMR: minimum redundancy maximum relevance; WRST: Wilcoxon rank-sum test				
**Feature selection**	**Machine learning classifiers**			
	**LDA**	**QDA**	**SVM**	**Random forest**
**mRMR**	0.70	0.74	0.76	0.79
**WRST**	0.75	0.78	0.78	0.81

Table S4. Classification performance (in terms of AUC-ROC) of each feature family using a Wilcoxon rank-sum test for feature selection and random forest as the classification algorithm

**Feature family**	**Description**	**UHCMC validation (n = 51 + 79 = 130)**	**TCGA validation (n = 143 + 54 = 197)**
Nuclei shape	Nuclear shape features	0.77	0.75
CGT	Entropy of nuclear orientation	0.70	0.66
cCCM	Local cellular diversity	0.75	0.70
Haralick	Texture features	0.58	0.43
CRL	Cell run length features	0.41	0.32
FeDeG	Feature-driven cell graph	0.53	0.48
Overall		0.81	0.78

Table S5. Thirteen Haralick measurements of the co-occurrence matrix (CM). The 'intensity' refers to the quantification levels of pixel intensity here, for example. $P_{i,j}$ represents the element in CM, where *i* and *j* represent the indices of the quantification level. P_x and P_y are the partial probability density functions. P_{x+y} is the probability of CM coordinates summing to *x* + *y*, respectively. N_g is the quantification level. $\mu_x, \mu_y, \sigma_x, and\ \sigma_y$ are the means and standard deviations of P_x and P_y, respectively.

Descriptor	Intuitive description	Equation
Entropy	Measure of randomness of CM values<br>High entropy: large variations in CM values extracted from an image<br>Low entropy: increasingly homogeneous CM values	$\sum_{i,j} P_{i,j} \log P_{i,j}$
Energy	Measure of homogeneity of CM values<br>High energy: increasingly uniform distribution of CM values from an ROI<br>Low energy: increasingly heterogeneous CM values	$\sum_{i,j} P^2{}_{i,j}$
Contrast	Measure of variations present in local regions of contrast<br>High value: higher spatial frequencies corresponding to large differences in a contiguous set of intensities<br>Low value: lower spatial frequencies	$\sum_i \sum_j (i-j)^2\ (P_{i,j})$

Information measure of correlation 1	Measure of linear dependency with respect to directional entropy. Roughly, it inversely varies with correlation but provides a more 'natural' measure of correlation due to its invariance under logarithmic transformation<br><br>High IMC1: greater presence of heterogeneous CM values in linear directions<br><br>Low IMC1: uniformity of CM values in linear directions	$\frac{\text{entropy} + \sum_i \sum_j P(i,j) \log\{P_x(i) P_y(j)\}}{\max(\text{entropy}_x, \text{entropy}_y)}$
Information measure of correlation 2	Measure of linear dependency with respect to randomness of spatial dependency. Roughly, it directly varies with correlation, but provides a more 'natural' measure of correlation due to its invariance under logarithmic transformation<br><br>High IMC1: higher presence of repeating patterns in linear directions<br><br>Low IMC2: lack of uniformity or repeating patterns in linear directions	$\sqrt{1 - \exp\left[-2.0\left[-\sum_i \sum_j P_x(i) P_y(j) \log\{P_x(i) P_y(j)\}\right]\right]}$
Intensity average	Measure of CM distribution relationship to mean intensity accumulations<br><br>High sum average: indicative of higher presence of punctate regions of high	$\sum_{i=2}^{2N_g} i P_{x+y}(i)$

	intensity<br><br>Low sum average: lack of presence of such punctate regions	
Intensity entropy	Measure of CM relationship to distribution of intensity with respect to entropy. High and low values correspond similarly to entropy values	$-\sum_{i=2}^{2N_g} P_{x+y}(i) \log\{P_{x+y}(i)\}$
Contrast entropy	Measure of CM relationship to intensity differences with respect to entropy<br><br>Inversely varies with intensity entropy	$\sum_{i=0}^{N_{g-1}} P_{x-y}(i) \log\{P_{x-y}(i)\}$
Contrast average	Measure of CM relationship to mean intensity differences.<br><br>Inversely varies with intensity average	$\sum_{i=0}^{N_{g-1}} iP_{x-y}(i)$
Correlation	Measure of intensity linear dependency<br><br>High correlation: scale of local pattern is larger than the distance<br><br>Low correlation: scale of local pattern is smaller than the distance	$\sum_{i,j} \frac{(ij)p(i,j) - \mu_x\mu_y}{\sigma_x\sigma_y}$
Intensity variance	Measure of CM relationship to distribution of intensity with respect to variance<br><br>High sum variance: greater standard deviation of sum average	$\sum_{i=2}^{2N_g} (i - \text{Intensity Entropy})^2 P_{x+y}(i)$

	Low sum variance: low standard deviation of sum average	
Contrast inverse moment	Measure of local regions of homogeneity<br><br>High value: higher presence of locally uniform windows in CM<br><br>Low value: higher presence of locally heterogeneous windows in CM	$\sum_{i,j} \frac{P_{i,j}}{1+(i-j)^2}$
Contrast variance	Measure of CM relationship to intensity differences with respect to variance<br><br>Inversely varies with sum variance	$\sum_{i=0}^{N_{g-1}} (i - \text{Sum Entropy})^2 \, P_{x-y}(i)$
